# Touch IoT enabled by wireless self-sensing and haptic-reproducing electronic skin

**DOI:** 10.1126/sciadv.ade2450

**Published:** 2022-12-23

**Authors:** Dengfeng Li, Jingkun Zhou, Kuanming Yao, Sitong Liu, Jiahui He, Jingyou Su, Qing’ao Qu, Yuyu Gao, Zhen Song, Chunki Yiu, Chuanlu Sha, Zhi Sun, Binbin Zhang, Jian Li, Libei Huang, Chenyu Xu, Tsz Hung Wong, Xingcan Huang, Jiyu Li, Ruquan Ye, Lei Wei, Zhengyou Zhang, Xu Guo, Yuan Dai, Zhaoqian Xie, Xinge Yu

**Affiliations:** ^1^ Department of Biomedical Engineering, City University of Hong Kong, Hong Kong SAR 999077, China.; ^2^ Hong Kong Centre for Cerebro-Cardiovascular Health Engineering (COCHE), Hong Kong SAR 999077, China.; ^3^ State Key Laboratory of Structural Analysis for Industrial Equipment, Department of Engineering Mechanics, Dalian University of Technology, Dalian 116024, China.; ^4^ Ningbo Institute of Dalian University of Technology, Ningbo 315016, China.; ^5^ Department of Chemistry, City University of Hong Kong, Hong Kong SAR 999077, China.; ^6^ Tencent Robotics X, Shenzhen 518054, China.; ^7^ City University of Hong Kong Shenzhen Research Institute, Shenzhen 518057, China.

## Abstract

Tactile sensations are mainly transmitted to each other by physical touch. Wireless touch perception could be a revolution for us to interact with the world. Here, we report a wireless self-sensing and haptic-reproducing electronic skin (e-skin) to realize noncontact touch communications. A flexible self-sensing actuator was developed to provide an integrated function in both tactile sensing and haptic feedback. When this e-skin was dynamically pressed, the actuator generated an induced voltage as tactile information. Via wireless communication, another e-skin could receive this tactile data and run a synchronized haptic reproduction. Thus, touch could be wirelessly conveyed in bidirections between two users as a touch intercom. Furthermore, this e-skin could be connected with various smart devices to form a touch internet of things where one-to-one and one-to-multiple touch delivery could be realized. This wireless touch presents huge potentials in remote touch video, medical care/assistance, education, and many other applications.

## Teaser

A wireless self-sensing and haptic-reproducing electronic skin enables a bi-directional touch communication as a touch intercom.

## INTRODUCTION

With the rapid development of virtual and augmented reality (VR/AR), information from visual and auditory senses is no longer sufficient for us to build immersive experience in the VR/AR applications (*1*–*5*). The absence of touch sense is the major hurdle for us to get more realistic feeling in the virtual scenarios as we get in the real physical world (*6*). In reality, we can feel each other’s touch very easily, but we cannot directly interact and communicate with the virtual world by touch because of the absence of physical contact. In view of these challenges and urgent demands, the perception and transmission of touch sensations are critical. With joining of the touch sense as the third major sensory perception, user experience in virtual life will predictably take a qualitative leap forward (*7*). However, at this stage, there is a considerable lack of studies on touch communication and concrete solutions to make touch sensations as pervasive in our lives as visual and auditory sensations (*8*).

Since skin is the organ for tactile sensation, the soft electronic skin (e-skin) would be an excellent type of device that fits conformably to the body for implementing tactile sensing and haptic feedback (*9*–*15*) without adding substantial burdens on our daily activities (*16*–*20*). For tactile sensing (*21*, *22*), a variety of materials and functional devices were used to detect external stimuli to collect information of the force and temperature at the time of being touched (*23*–*31*). For haptic feedback (*32*), active mechanical (*33*–*35*), electrical (*36*), and temperature (*37*) stimulations by the e-skin allow our skin to acquire the touch sensations from various scenes without having to touch them directly. Unfortunately, current e-skins only have a dull function with either tactile sensing or haptic feedback.

Similar to visual and auditory communication, touch communication in a wireless way allow the transmission of tactile sense among different users. One user that touches a tactile sensing e-skin can send “touch” information to another user who also wears a haptic feedback e-skin by feeling the haptic reproduction (*38*). However, this touch transmission is unidirectional only from the tactile sensing end to the haptic feedback end. To realize a bidirectional touch communication in the similar way as voice intercoms do, an e-skin with both tactile sensing and haptic feedback functions is mandatory (*39*). With the support of internet, we are able to further build a touch network to make touch sense function as a primary sense such as sight and hearing (*40*, *41*).

Here, we report a class of materials, devices, integration strategies, and wireless communication schemes for an intelligent self-sensing and haptic-reproducing e-skin in constructing wireless touch internet of things (IoT). On the basis of a range of material and structural designs, an electromagnetic-based flexible device can be developed with both tactile sensing and haptic feedback functions as a self-sensing actuator. The sensing function is derived from the induced current generated by the position change between the magnet and the coil as the device is deformed by the external force loading. The haptic feedback can be achieved by the mechanical tapping and vibrations of the devices. By applying an alternating current to the coil, the magnet underwent a periodic oscillation under the Lorentz force in the alternating magnetic field, which produces a mechanical stimulation to the skin. With a series of actuators assembled with scalable circuit designs in matrix, the integrated soft e-skin can be used for wireless tactile sensing and haptic reproduction. A bidirectional and networked touch transmission is achieved among multiple users via these e-skins. This concept and technology present a prototype for the construction of touch IoT, which is promising to be the cornerstone of the development of future touch network.

## RESULTS

### Design of the self-sensing and haptic-reproducing e-skin

**Fig. 1.**
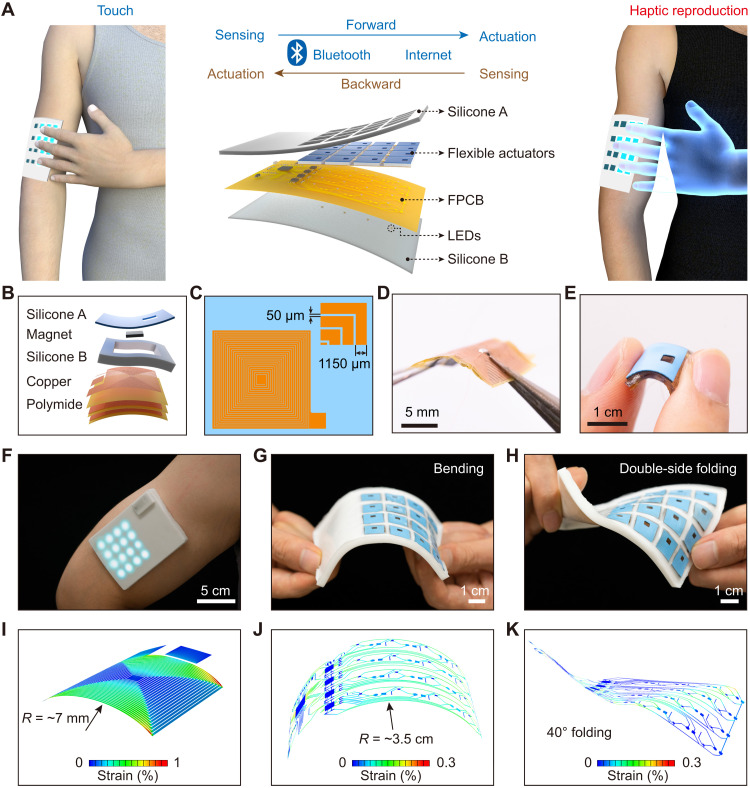
Design and architecture of a self-sensing and haptic-reproducing e-skin. (**A**) Schematic diagram of the wireless bidirectional touch transmission based on the self-sensing and haptic-reproducing e-skin system. This e-skin contains 16 flexible self-sensing actuators, 16 corresponding light-emitting diodes (LEDs), and a flexible printed circuit board (FPCB) with functions of wireless actuation and Bluetooth communication. (**B**) Exploded-view schematic of a flexible actuator with a flexible trilayer coil, a soft silicone support, a magnet, and a soft silicone film. (**C**) Diagram of a coil electrode. (**D** and **E**) Photographs of a flexible trilayer coil and a flexible self-sensing actuator. (**F**) A photograph of an e-skin fitted conformally on the arm with illuminated LEDs. (**G** and **H**) Photograph of an e-skin system under bending and double-side folding. (**I**) FEA results of a bending flexible coil. (**J** and **K**) FEA results of an e-skin system under bending and double-side folding.

To construct a wireless touch network for spaced touch transmission, we developed a self-sensing and haptic-reproducing e-skin. With the integrated wireless tactile sensing and haptic feedback array in the e-skin, the sense of touch can be synchronized and transmitted directly from one e-skin to another. Figure 1A shows the concept of the touch transmission, where two persons wear the e-skins for tactile information collection, transmission, and reproduction. When one person touches the e-skin, the perceived touching information is converted into a tactile electrical signal that can be wirelessly transmitted to the other e-skin via the Bluetooth. Once the tactile signal is received by the other e-skin, the corresponding haptic actuators are triggered to vibrate for the haptic reproduction to the other person. Conversely, this form of reversed touch transmission can also be implemented. This bidirectional touch transmission is analogous to the existing voice intercoms, so this touch communication can be called as touch intercoms. In this way, multiple touch intercoms and a touch communication network can be constructed on the basis of the e-skins, just like the current social networks through sight and sound.

This e-skin consists of a 4 × 4 array of flexible sensors/actuators, electronic components of a microcontroller unit (MCU), a Bluetooth module, and other electronics on a flexible circuit board (Fig. 1A). The flexible self-sensing actuator serving as the core part of the e-skin consists of a trilayer flexible coil, a soft silicone support, a magnet, and a thin polydimethylsiloxane (PDMS) film (Fig. 1B). The trilayer flexible coil stacks three single layer of copper (Cu) coil, which is fabricated by the photolithography and wet etching processes with an Cu-coated (18-μm-thick) polyimide (PI; 12.5 μm in thickness) (Fig. 1, C and D, and fig. S1). After assembling of all the individual components, a flexible 2.7-mm-thick square self-sensing actuator can be obtained (side length of 12 mm), where the square neodymium magnet serves as the vibrator (3 mm by 3 mm by 0.5 mm) (Fig. 1E and figs. S2 and S3A). Under AC power input, this actuator will generate a mechanical vibration and provide the skin with a touch sense. When this actuator is pressed by an external force, its flexibility allows it to deform and generate an electromagnetically induced voltage signal for tactile sensing.

The e-skin with a 4 × 4 self-sensing actuator array is as thin as 4.2 mm after accessories integration and encapsulation (Fig. 1F and figs. S3B and S4). This e-skin also has 16 corresponding light-emitting diodes (LEDs) to visualize the haptic feedback states. Figure 1 (G and H) demonstrates the softness of this e-skin under bending and double-side folding, which indicates the great wearable capability of this e-skin. Finite element analysis (FEA) simulation results show the strain distribution in the Cu layers of an individual flexible coil in the actuator under bending and the strain distribution in the Cu layers of the entire e-skin under bending and double-side folding (Fig. 1, I to K). The results represent that the entire flexible e-skin exhibits stable mechanical properties that can ensure a conformal fit to the skin and to maintain good working properties.

### Tactile sensing

**Fig. 2.**
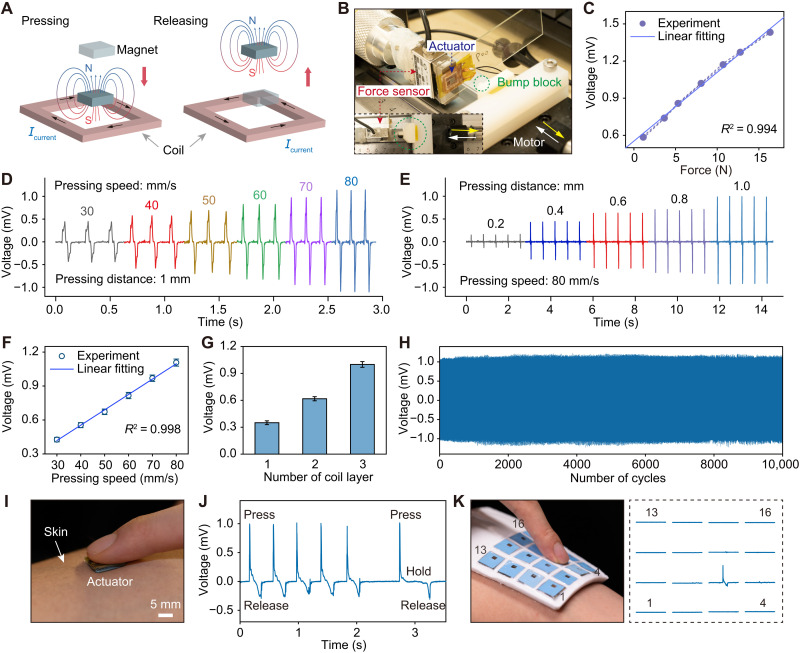
Tactile sensing performances. (**A**) Principle of sensing for flexible actuators based on Faraday’s law of electromagnetic induction. Alternating magnetic field produces an alternating electric field to induce a current in the coil during the pressing and releasing of the magnet. (**B**) Sensing test setup with a motor, a force sensor, a bump block, and an actuator from the side view and top view (inserted photograph). (**C**) Relationship between the sensing response voltage and the force generated by the dynamic press. (**D**) Sensing voltage at different pressing speed with a fixed pressing distance of 1 mm. (**E**) Sensing voltage under different pressing distances with a fixed pressing speed of 80 mm/s. (**F**) Linear relationship between the pressing speed and the sensing response voltage. (**G**) Sensing voltage for the actuators with different layers of coils. (**H**) Sensing cycle performances of the flexible actuator. (**I**) Photograph of pressing an actuator on the skin. (**J**) Sensing voltage of pressing the actuator on the skin. (**K**) Pressing an actuator on the e-skin array and the corresponding sensing distribution.

The tactile sensing function of the flexible self-sensing actuator is derived from Faraday’s law of electromagnetic induction. The sensing principle is based on the change rate of the magnetic flux related to the deformation speed. When the sensor is dynamically pressed with a pressing speed, the flexible self-sensing actuator deforms, and the relative distance between the magnet and the coil changes. As shown in Fig. 2A, the pressed magnet comes close to the coil, and a top-view clockwise induced current is generated. When the press is released, the magnet moves away from the coil, and a corresponding counterclockwise induced current is generated. The values of these electrical signals will offer important information of tactile strength and duration. By setting up a test system as shown in Fig. 2B, we can quantify the relationship between the external dynamical press and the tactile sensing voltage. A bump block with a cylinder (diameter of 4 mm) was mounted on a linear motor, and a 12-mm actuator was tightly pasted on a force sensor that had been fixed on the other end of the slide (fig. S5). The top cylinder of the bump block and the magnet in the actuator were aligned in the same line. The cylinder on the bump block and the magnet in the actuator were aligned in the same line. During the test, the actuator was connected to the data acquisition system (PowerLab 16/35), and the magnet was pressed and released by the block driven by a linear motor. Thus, the relationship between the loaded force generated by the dynamic press and the tactile sensing voltage was acquired, where the results show that sensors exhibit a good linearity with a *R*^2^ value of 0.994 (Fig. 2C). More intuitively, the sensing signal generated by electromagnetic induction is closely related to the pressing speed and distance between the magnet and the coil (Fig. 2, D to F). Figure 2D summarizes the changes in sensing voltage over time under different pressing speeds at a fixed pressing distance of 1 mm, which presents a signal-to-noise ratio (SNR) of 34.05 dB and a good linear relationship with a high *R*^2^ value of 0.998 between the pressing speed and sensing voltage (Fig. 2F). The sensing performance ensures good signal recognizability and predictability for tactile perception. The voltage signal waveform associates with pairs of positive/negative peak signals generated by the pressing/releasing magnet caused electromagnetic induction. When the pressing speed is fixed at a certain value, there is a positive correlation between the sensing voltage and the pressing distance (Fig. 2E and fig. S6), as the deeper pressing distance corresponds to greater change in magnetic flux and therefore a stronger electromagnetically induced voltage. Furthermore, the influence of the device size, magnet size, and coil turns on sensing voltage was investigated. Actuators with different coil sizes were first tested, where it was found that the sensing voltage increased with actuator size (fig. S7). The results also show that the 12-mm device exhibits both a strong sensing signal and relatively high-dimensional resolution compared to the 6- and 18-mm devices. As a result, we chose the device with a 12-mm side length for further demonstrations. Figure S8 shows that the sensing voltage also enhances as the magnet size increases. While given that large magnets inevitably affect the flexibility of the device, a 3-mm magnet was selected for the device, which is large enough to provide a sufficient sensing signal. Last, the sensing voltage shows a multiplicative increase with the number of turns of the coil, and the trilayer coil was selected (Fig. 2G). Thus, a 12-mm device with a 3-mm magnet and a trilayer coil was selected for subsequent experiments and applications. In the cycling performance test with 10,000 cycles, the sensing voltages show very stable properties (Fig. 2H). The average value of positive peaks is 1.092 V with an SD of only 0.038 V (fig. S9). Comparing with the reported tactile sensors with different working mechanisms, this electromagnetically induced self-sensing actuator in this work performs well in terms of linearity, SNR, and cycling stability (table S1) (*42*–*49*). It is worth mentioning that the self-sensing actuator also owns the function of haptic feedback.

To validate the potential for practical applications, we also tested the tactile sensing signal by placing the flexible actuator on the skin and pressing it by fingers (Fig. 2I). As shown in Fig. 2A, a positive pulse signal is generated when the actuator is pressed, and a negative voltage signal appears when the actuator is released (Fig. 2J). During the intermediate phase when the actuator is held down, the voltage signal is maintained at a level of around 0 V (Fig. 2J). When the device was placed on the skin, the sensing voltage may be influenced by the depression deformation of the soft skin. For the device sensing on the soft skin, the calculated sensing discrepancies will happen if we use the tested law on the rigid substrate. To accurately calculate tactile sensing, we need more laws on the soft skins to compensate the voltage discrepancies compared to the rigid substrate. Therefore, we added the test of the sensing performance of the device on 1-cm-thick artificial skin with the elastic modulus of 10 kPa (50:1 PDMS), 23 kPa (45:1 PDMS), 38 kPa (40:1 PDMS), 70 kPa (35:1 PDMS), and 135 kPa (30:1 PDMS). The tested sensing voltages on the artificial skin are smaller than that on the rigid substrate because the depression of the artificial skin causes a smaller relative pressing distance between the magnet and the coil. Moreover, slopes *k* of the sensing voltages with pressing speed on soft artificial skins decreases as the elastic modulus decreases (fig. S10B). If the device was used for sensing test on the skin with elastic modulus of *E*, then we could calculate the slope first according to the fitting equation in fig. S10C. Then, the tactile sensing expressed as pressing speed *v* can be accurately calculated by the equation of *v* = *U*/*k*, where *U* is sensing voltage. Meanwhile, we also investigated the interference signals of the sensors generated by the body movements. For general walking and jumping, the maximum peak values are only 0.0086 and 0.013 mV, respectively, which are far less than the 0.3- to 1-mV voltage range for general tactile sensing (Fig. 2, C and F, and fig. S11). We can therefore conclude that the effect of body movement would not cause interference or accuracy issue on the sensing characteristics of the device. Last, the integrated e-skin with an array of 16 self-sensing actuators was applied to the body to perform the tactile sensing test. When a particular actuator was pressed with a finger, this actuator presented a distinct tactile sensing signal (Fig. 2K). Given the slight deformation of the e-skin caused by the press, an adjacent actuator also generated a weak signal. The result reflected well the tactile sensing distribution because the press on the real skin would also cause a tugging sensation on the adjacent skin. After testing by the small bump block, a pressed magnet would not induce the adjacent actuator to generate a sufficient detectable signal since the size of the magnets is only 3 mm, and a central distance between adjacent actuators reached 14 mm. These results show that this e-skin performs well as a tactile sensing application.

### Haptic feedback

**Fig. 3.**
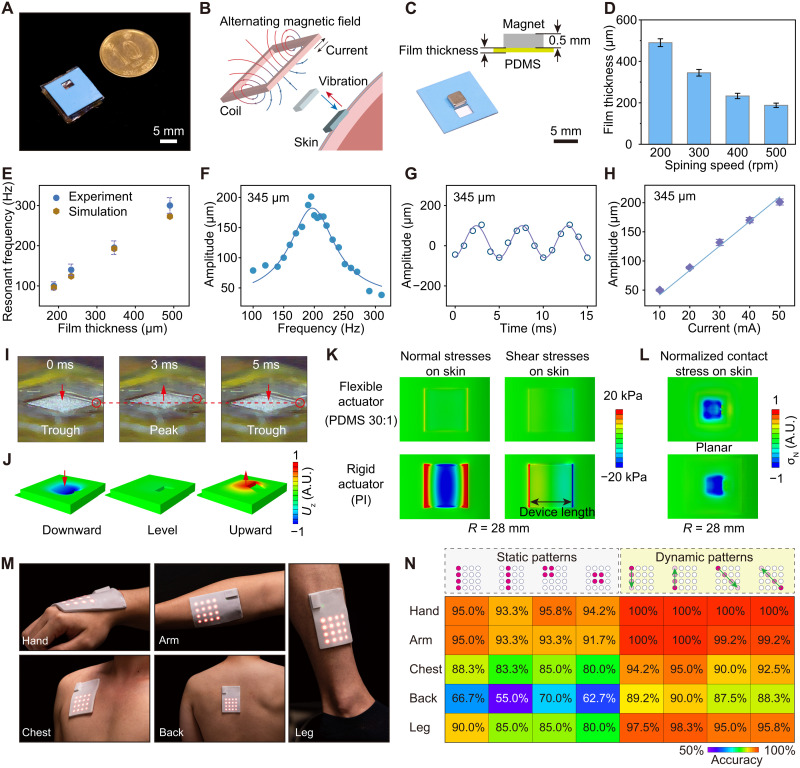
Haptic feedback performances. (**A**) Photograph of a flexible actuator and a 10-cent Hong Kong dollar coin. (**B**) The principle of haptic feedback by the flexible actuator. The oscillation of the magnet induced by the alternating electric field exerts mechanical feedback on the skin. (**C**) Different PDMS films with a 0.5-mm-thick magnet. (**D**) Relationship between PDMS thickness and spin-coating speed. (**E**) Resonant frequencies of the actuators with different thick PDMS films. (**F**) Amplitude at different actuation frequencies for the flexible actuator with a resonant frequency at 195 Hz. (**G**) Values of the vibration amplitude with time for the actuator with 345-μm-thick PDMS film. (**H**) Relationship between the actuator’s vibration amplitude and the actuation sinewave AC current. (**I**) Optical images of troughs and peaks state for the vibrating actuator recorded using a high-speed camera. (**J**) FEA results of the actuator’s vibration in the downward, leveled, and upward direction. (**K**) FEA results of a flexible actuator and a rigid actuator applied on the skin. The flexible actuator presents low stress to the skin. (**L**) The normalized contact stress (σ_N_) distributions at the interface between the skin and actuator applied on (top) undeformed skin and (bottom) *R* = 28 mm, respectively, as shown in movie S3. (**M**) Photographs of the self-sensing and haptic-reproducing e-skin on different body parts. (**N**) Accuracy summary of the haptic feedback test by four representative static patterns and four dynamic patterns applied to five typical parts of the body.

**Fig. 4.**
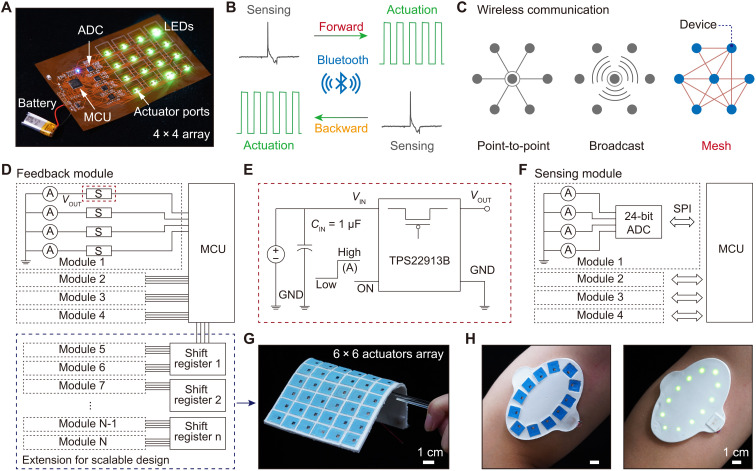
Wireless system of the e-skin for touch IoT. (**A**) Photograph of the flexible circuit for the wireless e-skin as a touch IoT device. (**B**) Forward and backward signal transmitting through Bluetooth. (**C**) Three common Bluetooth network topologies. (**D**) Circuit diagram of the feedback module for the haptic reproduction of the e-skin. The extension module is optional for the scalable design of the e-skin. (**E**) Schematic diagram of electric switch. GND: Ground. (**F**) Circuit diagram of the sensing module for tactile sensing of the e-skin. (**G**) A photograph of the soft e-skin with a 6 × 6 array of actuators. (**H**) Photographs of an elliptical shape e-skin with 12 actuators.

This flexible self-sensing actuator is comparable to a 10-cent Hong Kong dollar coin in size (Fig. 3A). Haptic feedback function of this flexible self-sensing actuator is also derived from Faraday’s law of electromagnetic induction. When an AC is applied, the flexible coil generates an alternating magnetic field (Fig. 3B). This magnet attached on the thin PDMS film produces a periodic oscillation under the Lorentz force of this alternating magnetic field. This periodic oscillation then induces mechanical feedback to the skin for the haptic reproduction. The haptic feedback performance of this flexible self-sensing actuator is adjustable and can be customized by the thickness of the PDMS film (Fig. 3C). A spin coating of PDMS (5:1) was applied at 500, 400, 300, and 200 rpm, and four corresponding PDMS films were obtained with thicknesses of 188, 233, 345, and 490 μm after curing (Fig. 4D). The flexible actuators assembled with these four films exhibit four resonant frequencies of 100, 140, 195, and 300 Hz, respectively, which well agrees with the computational FEA results (Fig. 4E). The vibrations of these four actuators were recorded using a high-speed camera, and their vibration states are summarized and presented in fig. S12 and movie S1. The results illustrate that the thicker the PDMS film is, the higher the resonant frequency of the actuator is. In general, the resonant frequency *f* relates to the effective stiffness *K*∝*Eh*^3^ and effective mass *M* of a vibrational system by *f* = (*K*/*M*)^0.5^, where *E* and *h* are the elastic modulus and PDMS thickness, respectively, and *M* is the magnet mass in this system since the PDMS film is very light compared to the magnet. Thus, with the same magnet, the resonant frequency of the actuator increases as the PDMS thickness increases. So, we can simply tune the resonant frequency of the actuator using PDMS handling layers with different thicknesses.

As our skin is most sensitive to mechanical vibrations in the frequency range from 100 to 300 Hz, we have therefore chosen the actuator with resonant frequency of ~200 Hz for the e-skin (*50*). As shown in Fig. 3F, the actuator exhibits the most intense vibration at its resonance frequency of 195 Hz with a vibration amplitude of 201 μm, corresponding to the maximum haptic feedback sensation. As the actuation frequency deviated away from the resonant frequency, the vibration amplitude of the magnet decreased. Such a change in vibration behavior is the same for the actuators with resonant frequencies of 100, 140, and 300 Hz and maximum amplitude values of 606, 245, and 60 μm at their resonant frequencies (fig. S13). All test results were based on the input of a 50-mA sinewave current to the actuator. As shown in Fig. 3G and fig. S14, the waveform of all actuators during an actuation cycle exhibits a sinusoidal shape, which is consistent with the waveform of the actuation current. Moreover, there is a linear relationship between the input current and the vibration amplitude of the magnet in the actuator (Fig. 3H).

During the actuation process, we captured the peak and trough moments in a cycle to show the displacement of the actuator’s magnet (Fig. 3I). Using FEA simulations, we also simulated the states of the actuator under a vibration cycle, with the magnet in downward, leveled, and upward positions (Fig. 3J and movie. S2). When the actuator is applied with the magnet side to the skin, the magnet vibrations produce haptic feedback to our body. Furthermore, the flexible actuator tends to own superior wearability characteristics compared to traditional rigid actuators (*2*, *33*, *34*, *50*). To test this hypothesis, we took an FEA simulation approach to presenting the difference between flexible and rigid actuators in the wearable situation. The flexible and rigid actuators were equivalent to a soft PDMS (30:1) and a PI material square sheet, respectively (see the simulation details in fig. S15). When the device is applied to the skin surface at a bending radius of 28 mm, the simulation results show that the rigid actuator exerts notable normal and shear stresses on the skin, while the soft flexible actuator does not exert notable stresses on the skin (Fig. 3K). When viewed from the side, the surface and deeper layers of the skin are subjected to high levels of stress on both sides of the rigid actuator (fig. S16). Therefore, the flexible self-sensing actuators are more comfortable and particularly suitable for wearable e-skins, especially for body surfaces with greater curvature. To verify whether the actuator can vibrate effectively on the curvilinear skin for the soft actuator, the normalized contact stress (σ_N_) distributions at the interface between the skin and actuator attached on undeformed skin and bending skin with a curvature radius of *R* = 28 mm were simulated. The results show that the contact stresses on the undeformed skin and curved skin have no significant difference for the soft actuator (Fig. 3L). FEA results of the dynamic vibrations for an actuator mounted on undeformed skin and bending deformed skin both show a smooth vibrational state (movie S3).

The e-skin integrated with these flexible self-sensing actuators can be applied closely to various parts of the body, such as the hands, arms, chest, back, and legs (Fig. 3M and fig. S17). Previous studies have implied that different parts of the body perceived and recognized haptic feedback differently (*51*). To investigate the ability of these body parts to recognize the haptic reproduction of this e-skin, a series of user experiments were carried out. We started with nine static patterns with four actuators working, including four single rows of line patterns in a single row and five square patterns in the four corners and a center (fig. S18). After putting the e-skin to different parts of 12 individuals’ bodies and controlling the e-skin to perform different patterns of vibrations, the individuals were asked to recognize the vibration patterns. As a result, the hands and arms demonstrated over 90% accuracy, and then the legs and chests were near 85% accuracy, while the back was only about 60% accurate (Fig. 3N). These results suggest that the upper limbs show a higher sensitivity and recognition of the haptic feedback from this e-skin, while the back shows the relatively lower sensitivity. The back skin of body can also clearly distinguish the haptic position difference from switching the operation of the adjacent actuators. It is difficult for the back to remember all perceived positions and build an accurate spatial pattern even if the skin sensed the spatial differences, while dynamic vibration switching gave more spatial and temporal information in a short period of time to improve the accuracy of haptic recognition (*52*–*55*). We added four dynamic patterns for the haptic tests (fig. S19). The results show that the average recognition accuracy of the dynamic patterns by the back reached 88.75% with an improvement of 20% that of the static pattern of 63.6%. In other parts of the body, this accuracy rate remains above 95%, ensuring that this e-skin is sufficient for daily haptic feedback use. The above results and tests provide a comprehensive overview of the applicable haptic reproduction capabilities of this e-skin.

It is difficult to accurately recognize the complex patterns through skin perception compared to eyes, so we selected nine representative static and dynamic patterns for user experiments, although this e-skin can provide more patterns (65,535 patterns in theory). It is more likely for our skin to perceive the positional and dynamic directional tactile information via simple patterns. Therefore, our goal tends to communicate precise information based on simple patterns, to transmit vague touch sense based on complex patterns through this e-skin, and to provide an additional means of remote communication besides the visual and auditory senses.

### Wireless system of the e-skin

A wireless communication approach is applied in this e-skin system to achieve the touch transmission across space. Figure 4 summarizes the entire wireless system with circuit modules in tactile sensing and haptic feedback, the touch transmission, and the touch networking. As shown in Fig. 4A, the flexible circuit of the e-skin consists of an MCU with a built in Bluetooth module, four 24-bit analog-to-digital converters (ADCs), 16 flexible self-sensing actuators, 16 electronic switchers, and 16 LEDs. More details of the circuit diagram design and circuit photos are displayed in the fig. S20. The electrical signals generated from the self-sensing actuators corresponding to the external dynamic press are converted to digital signals by the ADCs and sent to the MCU via Serial Peripheral Interface. The e-skin operates in tactile sensing mode by default. When an e-skin experiences an external dynamic pressing, it will send this tactile information out via Bluetooth. When other e-skins receive the tactile signal, the corresponding actuators will be activated and switched to haptic feedback mode for haptic reproduction, and vice versa (Fig. 4B). For the e-skin for haptic reproduction test, the actuation voltage on the actuator is a positive 2.8-V square wave with a skin-sensitive related frequency of 195 Hz (fig. S21). Although the soft film dampen the vibrations slightly compared to the rigid film, the soft actuator can still generate a force of 12.1 mN that enables to provide an obvious vibration feeling to the human skin, as which is much greater than the skin’s sensation threshold (fig. S22) (*56*). A 200-μm vibration displacement at 200 Hz for the flexible actuator also ensures that its feedback is enough for tactile perception because the minimum threshold of displacement for the glabrous skin is as small as 0.1 μm at 150 to 300 Hz (*53*). Compared to rigid eccentric rotating mass and linear resonance actuator, the haptic feedback intensity of this flexible actuator is weaker, but it still has two obvious advantages: the functions of tactile self-sensing and a wide frequency resonance peak with the half height width of ~100 Hz (table S2) (*34*, *57*). At a very low actuation voltage of 2.8 V, this flexible actuator has a comparable level of feedback intensity to the flexible piezoelectric and piezoelectret actuators (table S2) (*35*, *58*). In contrast, several hundred volts for piezoelectric actuation is bound to present some difficulties in wireless circuit design. The power consumptions of the Bluetooth control circuit and the actuator are 23.1 and 115.3 mW, respectively. Some strategies can be adopted to decrease the power consumption of the actuator. The size of the magnet can be increased to enhance the electromagnetic force of the magnet. In this way, the power requirement will be lowered for the same feedback strength. In addition, the layer number of the coil can be increased to enhance the magnetic flux density B with each layer being 23 turns of copper wire. As shown in fig. S23, as the layer number increases, the magnetic flux density of the coil increases rapidly and then decreases gently under the constant actuation power. When the layer number of the coil increases to 75 layers, the magnetic flux density reaches a maximum value. The power consumption of the actuators with 75 layers of coil can be lowered to 14 mW when maintaining the same feedback strength.

There are several ways to transmit data between multiple Bluetooth devices, namely, point-to-point, broadcast, and mesh network (Fig. 4C) (*59*). Compared to the first two topologies, mesh networks have larger communication distance, more connected nodes, increased reliability, and greater responsiveness with node-to-node communication (*60*). To form the wearable touch IoT system, we connect several of the above-mentioned e-skins into the Bluetooth mesh network. The events sent by any e-skin in the network can be received by other e-skins in the network, thus making it feasible for touch to be transmitted in the network similar to video and audio. When e-skin works under the haptic feedback mode, the self-sensing actuator works as a linear resonant actuator to provide vibration feedback. There are 16 actuators along with corresponding electronic switches in the feedback module (Fig. 4, A and D). The electronic switch contains a P-channel metal oxide semiconductor field-effect transistor that can be controlled by an on/off input to the gate electrode (Fig. 4E). The on/off state of the electronic switch can be controlled by the low-voltage pulse-width modulation (PWM) signals output from the general-purpose input/output (GP I/O) in the MCU to drive the actuators for haptic feedback. Meanwhile, 16 LEDs are used to indicate the actuators’ operating status for visualization. When e-skin works under the tactile sensing module, the self-sensing actuator works as a magnetic induction sensor. The tactile sensing module shares the 16 self-sensing actuators with the haptic feedback module, and additional four four-channel ADCs are included (Fig. 4F). In this circuit, the flexible device acts as both the tactile sensor and actuator. The use of a multifunctional device greatly simplifies the circuit design comparing to the platform with separate sensor and actuator modules. Furthermore, the number of input/output ports and connections in the circuit can be significantly reduced.

The e-skin can operate in tactile sensing and haptic feedback modes simultaneously for different actuators on one e-skin, but tactile sensing and haptic feedback cannot be operated at the same time of one actuator. When an actuator in the first e-skin is pressed and generates a sensing signal, the haptic feedback mode for this actuator is switched off. When the second e-skin receives the sensing signal from the first e-skin, the corresponding actuator is switched from the default tactile sensing mode to the haptic feedback mode. At this moment, this actuator is not able to perform the tactile sensing function when it is vibrating. However, since the wireless circuit allows for simultaneous transmission of touch signals in bidirections, other actuators on the second e-skin can still perform tactile sensing functions. That is, we can press other actuators of the second e-skin to send tactile transmissions to the first e-skin. This bidirectional touch transmission can operate in the form of multiple tracks between two e-skins. Therefore, this bidirectional tactile sensing and haptic feedback can be achieved simultaneously.

This wearable e-skin is scalable in the matrix design. The e-skin matrix size can be easily expanded for bigger coverage area by grouping more 4 × 4 arrays together. Touch communication can be realized through the bidirectional transmission between the corresponding arrays on user 1 and projected arrays on user 2. This e-skin can also be expanded by increasing the number of actuators in a single array to provide a larger area and higher resolution haptic feedback. As an example, Fig. 4G shows an expanded 6 × 6 actuators array based on one MCU. The circuit design method we used is also universal for even larger actuator arrays, which can be easily realized by adding an extension module. The extension module is composed of multiple shift registers connected in series, which only occupies four input/output ports of the MCU, and each shift register can control eight actuators (Fig. 4D and fig. S24). By connecting multiple shift registers in series, it can control a large number of actuators at the same time. The maximum number of the pixels in a single array is limited by the number of GP I/Os and the operating frequency of the MCU. In addition, this scalable e-skin can be designed into an elliptical shape or other different shapes for a better fit on different parts of the body (Fig. 4H and fig. S24).

### Applications of the e-skin for touch-IoT

**Fig. 5.**
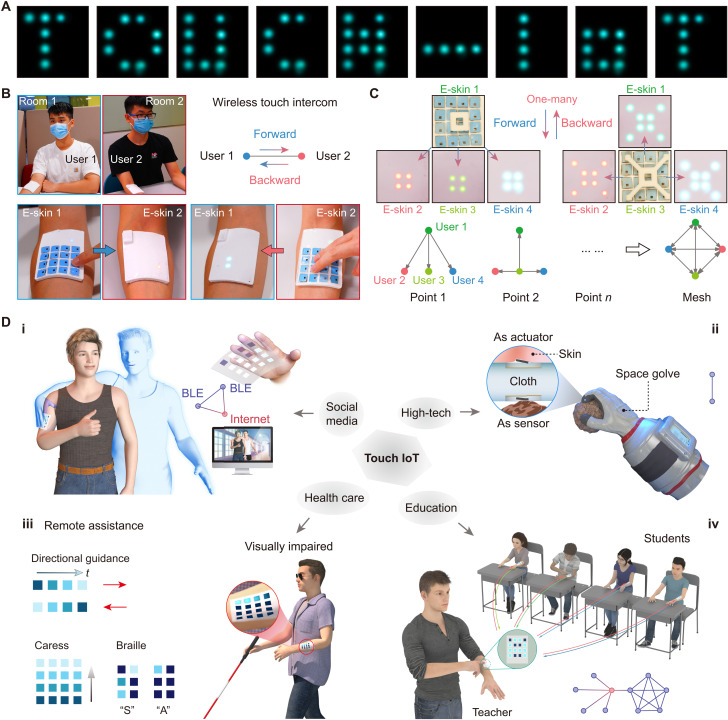
Examples of applications of the self-sensing and haptic-reproducing e-skin for touch IoT. (**A**) Haptic feedback patterns of the letters “T” “O” “U” “C” “H” “-” “I” “o” “T” by the e-skin. (**B**) Attaching two devices to the arms of two users for point-to-point wireless touch intercom in two rooms. (**C**) A Bluetooth touch mesh network formed using four e-skins. The touch detected by any one e-skin can be reproduced on the other three users by the e-skins. The diagram (below) briefly depicts the Bluetooth mesh signal transmission. (**D**) Touch IoT’s possible applications in future emerging fields of social media (i), high technology (ii), health care (iii), and education (iv).

A touch IoT can be built among multiple users by wearing these e-skins that allows the touch sense to be networked in the same way as hearing and sight. Each e-skin contains 16 actuators in a 4 × 4 array that can theoretically perform 65,535 static vibration patterns. With PWM signals controlling the amplitude of the actuators, an infinite number of vibration combinations can be produced. As an example, Fig. 5A, movie S4, and fig. S25 display the vibration patterns of characters “TOUCH-IoT,” “BLUETOOTH,” and 10 Arabic numerals. To demonstrate the practical application, a scene was built where two users sit in two separate rooms with our e-skins attached to their arms (Fig. 5B and movie S5). When user 1 pressed the e-skin on his arm, user 2 could feel the touch from user 1 via the e-skin. In turn, user 2 could also respond to user 1 with such a wireless touch. Since the self-sensing actuator integrates tactile sensing and haptic feedback functions, it can be used as both a sensor and an actuator. With the same e-skin, tactile signals can be transmitted between users, and the spaced-out touch can be felt by each other via the haptic reproduction, which we call a wireless touch intercom. With Bluetooth mesh network technology, touch is not limited to the transmission between two e-skins. In theory, Bluetooth mesh can connect tens of thousands of e-skins to work simultaneously. Figure 5C shows a Bluetooth mesh network with four end points. When e-skin 1 was pressed by a printed block, e-skins 2, 3, and 4 entered the haptic feedback mode to output haptic reproduction with four lighted LEDs for touch displays in a square shape, and when e-skin 2 was pressed by another block, e-skins 1, 3, and 4 then switched into the haptic feedback mode (fig. S26 and movie S6). In this way, a one-to-many touch network can be realized. Since there is no master or slave device in the Bluetooth mesh network, any e-skins that get disconnected will not affect the normal operation of the network.

Figure 5D illustrates several application scenarios of the touch IoT. Benefiting from the fact that the Bluetooth mesh network is based on Bluetooth Low Energy (BLE) (*61*), the e-skin can communicate with the smartphone or personal computer with BLE module directly and transmit data through the internet with those smart devices, which enable ultralong-distance touch transmission. Friends and family in different places can use the e-skin as touch IoT system to feel the cross-distance touch. In this way, the touch sensory dimension is directly added to existing video- and audio-based social media. As the third major sensory system, the addition of touch sense can greatly enhance the immersion of social media and shorten the distance among people. Another application is in high-tech fields such as on space where the touch sensation is often hindered, because the thick space suit inevitably hinders the direct contact between the astronaut’s skin and the external objects (*62*). This problem can be effectively solved using two of our e-skins that can be customized into specific shapes according to the design of the space suit. With one e-skin close to the skin and the other on the outside of the space suit, touch sensations can be transmitted between the two e-skins and reproduced to the astronaut so that the astronaut can feel the touch interaction with the outside, even if the astronaut’s skin is not in direct contact with external objects. In the health care field, the touch IoT enables a more realistic assistance and telemedicine experience (*63*–*65*). Visually impaired persons can wear this e-skin to gain remote directional guidance, to caress, and for Braille messages. They can also use this touch communication to call for help. Besides, in isolation wards especially under the current pandemic of coronavirus disease 2019, this haptic device can facilitate a realistic remote nursing with a lower risk of infection. Last, in the current educational context, online classes have become prevalent. Introducing touch into online or offline education would be a revolutionary innovation (*66*). Wearing the e-skins, teachers can deliver touch to all students in remote classes through the internet or in a physical classroom through the BLE. Students and teachers can communicate simultaneously by voice, video, and touch, which will greatly improve the educational experience.

At present, vibration actuator is an effective and indispensable tool for haptics (*32*, *67*), where the force feedback are typically based on the format of mechanical vibrations on the skin, which associates with alternative force oscillation of the actuator to create haptic feedback. When our skin is touched by the fingers in a sliding motion, a dynamic force is created. The skin haptic interface consisting of an array of vibrating actuators enables to provide very clear directional and sliding sensations in dynamic touch sense (*68*).

## DISCUSSION

In conclusion, we report a comprehensive strategy to build a touch network using a self-sensing and haptic-reproducing e-skin system. With such a touch network, users can feel others’ touch via the e-skin without direct physical contact with each other, which benefits from the integrated functions of an e-skin in tactile sensing and haptic reproduction. With a range of materials and structural designs, the core flexible self-sensing actuator implemented external dynamic press sensing based on the power generator principle and mechanical vibration feedback based on the motor principle, following Faraday’s law of electromagnetic induction. The integrated e-skin fitted on several parts of the body presents a stable touch transmission effect. This touch communication can take the form of a touch intercom between two people or for one-to-many touch among multiple people. With the Bluetooth, the e-skin can be also connected to a wide range of smart devices and relies on the internet for remote touch communication (*59*). In this way, a touch IoT can be built, enabling people to enjoy a touch communication experience just as voice calls and video calls. The touch IoT based on this e-skin could be widely used in social media, high technologies, health care, education, etc. This form of touch overcomes the limitations of space and is a huge advance that will greatly reduce the sense of distance in human communication.

## METHODS

### Fabrication of the flexible coil

Flexible coils were prepared on the basis of the processes of photolithography and wet etching. First, a thin copper-clad PI film (18-μm-thick Cu and 12.5-μm-thick PI) was flattened on a glass substrate with dimensions of 75 mm by 75 mm and cured at 75°C for 30 min. The PDMS (prepolymer and cross-link agent = 10:1; Sylgard 184, Dow Corning) served as the binding agent. Second, a positive photoresist (AZ-5214, AZ Electronic Materials) was spin-coated on the copper at 3000 rpm for 30 s and soft-baked at 110°C for 3 min. After exposure under ultraviolet (wavelength of ~350 nm) with a designed mask and development in AZ300 MIF developer, the copper film was wet-etched into the desired pattern in a FeCl_3_ solution. Last, the patterned copper-clad PI film was cut into square single-layer sheets with a laser-cutting machine (ProtoLaser U4, LPKF Laser & Electronics). By connecting the electrodes of three sheets in a clockwise direction and bonding those using PDMS, a multilayer bendable flexible coil was prepared.

### Fabrication of the flexible actuator

A flexible actuator consists of a multilayer flexible coil, a soft support, a PDMS film, and a magnet. The soft square-shaped support was fabricated from a cured PDMS (prepolymer and cross-link agent = 30:1) with a thickness of 2.2 mm. The PDMS films with different thicknesses were spin-coated from 5:1 PDMS at 200, 300, 400, and 500 rpm for 30 s. With laser cutting, these soft structures were all machined into designed shapes. A square neodymium 50 magnet (M0350, SuperMagnetMan) with dimension of 3 mm by 3 mm by 0.5 mm was fixed to the flexible film relying on the bonding and curing of PDMS. After assembling the coil, the support, and the film in alignment, a flexible bendable actuator was obtained.

### Circuit modules

To make the circuit flexible while having good mechanical stability, a thin flexible printed circuit board (FPCB) is fabricated with mechanical support layers and conductive layers. The FPCB is 139 μm in thickness and consists of top and bottom overlays made of PI film (27.5 μm in thickness), two patterned copper conductive layers (17.5 μm in thickness), a PI film (25 μm in thickness) to separate the two copper conductive layers, and two adhesive layers (12 μm in thickness) to bond the top and bottom overlay layers with the copper conductive layer. Electronic components including capacitors, resistors, inductors, LEDs, crystals, 2.4-GHz impedance-matched balun (2450BM14G0011, Johanson Technology), 2.4-GHz Mini Antenna (2450AT18A100E, Johanson Technology), MCU (CC2652R, TI), ADCs (ADS1220, TI), electronic switches (TPS22913B, TI), etc. are soldered on the exposed pads of the FPCB using low-temperature solder paste. The MCU with built-in BLE module can communicate directly with other Bluetooth devices through the 2.4-GHz antenna. The two pins of each flexible actuators are soldered to the pads reserved on the FPCB by fine copper wires, with one end of the two pins grounded and the other end connected to the 24-bit ADC input pin and the electronic switch simultaneously. When in sensing mode, the electronic switch is turned off, and the MCU continuously reads data from the ADCs to determine whether there is a dynamic press signal on the actuator; and when the voltage generated by the actuator is greater than the set voltage threshold, the MCU sends a command via Bluetooth. The command contains the index of the actuator that received the dynamic press signal, the address of the device that need to be turned on the feedback mode, etc. When the MCU receives feedback command from other devices, it switches to feedback mode, turns off the ADCs, and outputs PWM to the corresponding electronic switch according to the command, so that the flexible actuator starts to vibrate, and since the LEDs and electronic switches are connected in parallel, the corresponding LED will also light at this time. When the feedback is finished, the MCU switches back to sensing mode and waits for the next command or dynamic press signal.

### Fabrication of the self-sensing and haptic-reproducing e-skin

The preparation of the self-sensing and haptic-reproducing e-skin includes mold fabrication, soldering and encapsulation. A plastic mold was designed according to the size of the FPCB and was printed using a three-dimensional (3D) printer. A thin layer of parylene C was sputtered onto the plastic mold to prevent the PDMS from not curing during the subsequent encapsulation process. Before encapsulation, 16 flexible actuators were soldered on the prepared FPCB board with various electronic components and a battery. PDMS (prepolymer and cross-link agent = 30:1) and white dye were mixed as the encapsulation materials. For encapsulation, a suitable amount of uncured encapsulation material was first poured into the mold, and then the FPCB with actuators upward was placed in. Followed by vacuuming to remove any excess air bubbles and heating at 70°C for 30 min, the encapsulation material under the FPCB was cured. Then, additional encapsulation material was further poured into the mold to realize the encapsulation of the upper part of the e-skin. After removing the device from the mold, a soft and skin-friendly e-skin was created.

### Tactile sensing characterization

The sensing function of this flexible actuator was measured using a linear motor device (LinMot Ltd.) that can be precisely controlled. A force sensor was mounted on the top of the motor to detect the dynamic pressing force during the test. The flexible actuator was fixed to one end of the guide rail, and the 3D printed bump block was mounted at one end of the motor. The motors are set at different stepping distances and speeds to achieve different levels of impact, thus simulating different levels of touch. The voltage signal generated by the flexible actuator is detected in real time by the PowerLab 16/35 (PL 3516, AD Instruments). Last, to verify the durability of the flexible actuator, a 10,000 cycle test was carried out.

### Haptic feedback characterization

The haptic feedback of individual actuators was characterized by the vibration amplitude of the magnets. The actuators were powered by an AC source (Model 6221, Keithley Instruments Inc.) with sinewave current. During the actuation, the real-time vibrations of the flexible actuators were recorded by a high-speed camera (Keyence Corporation of America). The resonant frequency of the flexible actuators with different PDMS film thicknesses depends on the amplitude distribution calculated on the basis of the recorded video.

### Mechanical simulation of the e-skin

The FEA using commercial software Abaqus was used to study the mechanical characteristics of the flexible coil in the actuator and the e-skin. The strain distributions in the copper layers were discussed for the actuator under bending deformation (Fig. 1I) and the e-skin under bending and double-side folding deformations (Fig. 1, J and K). PDMS (30:1) and electronic components were modeled by hexahedron elements (C3D8R). The thin copper layers and PI layers were modeled by composite shell elements (S4R). The minimal element size was one-fourth of the width (150 μm) of Cu wires, which ensured mesh convergence and simulation accuracy. In the FEA, PDMS was assigned as hyperelastic materials using Mooney-Rivlin energy potential model, while other materials were assigned by elastic behaviors. The elastic modulus (*E*) and Poisson’s ratio (*v*) used in the analysis were *E*_Cu_ = 119 GPa and *v*_Cu_ = 0.34 for Cu; *E*_PI_ = 2.1 GPa and *v*_PI_ = 0.34 for PI; *E*_component_ = 10 GPa and *v*_component_ = 0.34 for electronic components; and *E*_PDMS-1_ = 145 kPa and *v*_PDMS-1_ = 0.49 for PDMS (30:1).

### Dynamic mechanics simulation of the actuator

Vibration behaviors of actuators were studied by modal analysis in commercial software Abaqus. The magnet and PDMS (30:1) layers were modeled by hexahedron elements (C3D8R), while thin PDMS (5:1), PI, and Cu layers were modeled by shell elements (S4R). The elastic modulus (*E*), Poisson’s ratio (*v*), and density (ρ) were *E*_PDMS-2_ = 3 MPa, *v*_PDMS-2_ = 0.49, and ρ_PDMS-2_ = 960 kg/m^3^ for PDMS (5:1); *E*_PDMS-1_ = 145 kPa, *v*_PDMS-1_ = 0.49, and ρ_PDMS-1_ = 960 kg/m^3^ for PDMS (30:1); and *E*_magnet_ = 113GPa, *v*_magnet_ = 0.34, and ρ_magnet_ = 7550 kg/m^3^ for magnet.

### Wireless touch network

Bluetooth mesh is a mesh network protocol based on the BLE protocol, which can connect local nodes to each other directly or indirectly so that information can be transmitted between any two nodes. In addition, since Bluetooth mesh is based on the BLE protocol, the smart phone can be used as a node to access the mesh network and send commands. To establish a Bluetooth mesh network, a smart phone is needed to work as a provisioner, and then each device will be connected to the phone for provisioning; when the establishment of the mesh network is completed, network can operate without the phone. In the Bluetooth mesh network, all the devices are worked as the proxy node to receive and retransmit mesh message. The MCU works in sensing mode by default; when an actuator receives a dynamic press signal, the MCU will send a mesh message to the Bluetooth mesh network; the mesh message contains the address of the device that sent the message, the address of the target device, the index of the actuator that received the dynamic press signal, and other information. By sending a mesh message to the Bluetooth mesh network, it is possible to control single or multiple devices for haptic feedback. Since the mesh network does not have a master-slave relationship, any node can send messages to the mesh network, and these messages can be sent simultaneously. When the target device receives the mesh message, it will drive the corresponding actuator for haptic feedback according to the content of the message. Bluetooth mesh network can access thousands of nodes at the same time, far more than the ordinary Bluetooth communication of eight devices and can forward mesh message by inserting relay nodes, so that the communication distance of mesh network is far greater than that of ordinary Bluetooth.

### User study of haptic feedback

An e-skin with 16 flexible actuators in a 4 × 4 array was chosen to conduct the user study to test the human body’s ability to recognize different haptic patterns. Four actuators were actuated simultaneously in the form of a 1 × 4 line or a 2 × 2 square to generate nine static patterns as shown is fig. S17. Four actuators were actuated sequentially with an interval time of 0.5 s in the form of a 1 × 4 straight line and a 1 × 4 diagonal line to generate four dynamic patterns as shown in fig. S18. The e-skin was also placed on various parts of the body of the individuals, including arm, chest, back, hand, and leg. Twelve individuals participated in this test. At a given body part of each individual, nine static actuation patterns and four dynamic patterns of the e-skin were randomly activated with each pattern appearing 10 times. One pattern on one part of the body corresponded to 120 groups of test data. Thus, 13 patterns with 7800 groups of data corresponding to five body parts and 13 haptic patterns were acquired to calculate the accuracy by comparing the individual’s perceived pattern and actual pattern. All user experiments follow ethical requirements.

### Application demonstration of wireless touch IoT

Four e-skins were fabricated with different light-up colors to demonstrate the wireless touch communication and touch IoT networks. First, two of the e-skins were used to display the one-to-one bidirectional touch transfer. Two individuals each worn an e-skin and sit in two different rooms. When a person taped the e-skin on his arm, the tactile information was sensed by the flexible actuators. Through the Bluetooth transmission, the e-skin in the other room would receive this tactile signal and then cause the corresponding actuators to vibrate. Thus, a wireless touch communication with haptic sensing and feedback was achieved. Likewise, the reverse communication can be implemented in the same way. This one-to-one approach corresponds to one-to-one spaced tactile intercom, neonatal intensive care unit care, and touch transfer for thick clothing. One-to-many bidirectional touch transfer and communication were also carried out using four e-skins. When one e-skin was pressed by a specific shaped printed block, the touch was wirelessly transmitted from this e-skin to the other three. Similarly, one of the other three e-skins can, in turn, transmit the sense of touch to the remaining three e-skins. In this way, a network of touch IoT was created. The application is analogous to a classroom where one teacher corresponds to multiple students, and the touch can be delivered by the teacher to all students at the same time. One of the students can also pass on the touch to the teacher and to the other students.
