# Supplementary material for: Touch IoT enabled by wireless self-sensing and haptic-reproducing electronic skin
Source: Sci Adv. 2022 Dec 23;8(51):eade2450. doi: 10.1126/sciadv.ade2450 (PMC9788763; doi:10.1126/sciadv.ade2450)
Supplement: Supplementary file 1 — Tables S1 and S2 Figs. S1 to S26 [file sciadv.ade2450_sm.pdf]

Science Advances
AAAS

Supplementary Materials for

Touch IoT enabled by wireless self-sensing and haptic-reproducing electronic skin

Dengfeng Li *et al.*

Corresponding author: Yuan Dai, jessiedai@tencent.com; Zhaoqian Xie, zxie@dlut.edu.cn; Xinge Yu, xingeyu@cityu.edu.hk

Sci. Adv. **8**, eade2450 (2022)
DOI: 10.1126/sciadv.ade2450

The PDF file includes:

Tables S1 and S2
Figs. S1 to S26
Legends for movies S1 to S6

Other Supplementary Material for this manuscript includes the following:

Movies S1 to S6

Table S1. Comparison of the tactile sensors with different working mechanisms.

Working mechanism	Linearity	SNR	Stability	Haptic feedback	Reference
Piezoresistive effect	$R^2 = 0.995$	N/A	>100000 cycles	No	*(42)*
Piezoresistive effect	N/A	20 dB	>6000 cycles	No	*(43)*
Capacitive effect	$R^2 = 0.99$	N/A	>45000 cycles	No	*(44)*
Capacitive effect	N/A	88.51 dB	>5000 cycles	No	*(45)*
Triboelectric effect	$R^2 = 0.99$	N/A	>250000 cycles	No	*(46)*
Triboelectric effect	N/A	8 dB	N/A	No	*(47)*
Piezoelectric effect	$R^2 = 0.972$	N/A	>7000 cycles	Yes	*(48)*
Piezoelectric effect	N/A	40.35 dB	N/A	Yes	*(49)*
Electromagnetic induction	$R^2 = 0.994$	34.05dB	>10000 cycles	Yes	This work

References are listed in the main text.

Table S2. Comparison of the haptic feedback performances of different type of actuators.

Type of actuators	Actuation voltage	Feedback force	Frequency	Power consumption	Tactile sensing	Reference
Rigid eccentric rotating mass	2.7V~3.3V	0.15G	NA	160mW	No	*(34)*
Rigid linear resonance actuator	1.8V	1.28G	235Hz	117mW	No	*(57)*
Flexible piezoelectric actuator	175V	NA	100Hz	375mW	Yes	*(58)*
Flexible piezoelectret actuator	495V	20mN	100Hz	NA	Yes	*(35)*
Flexible voice coil actuator	2.8V	12.1mN	100Hz-300Hz	115mW	Yes	This work

References are listed in the main text.

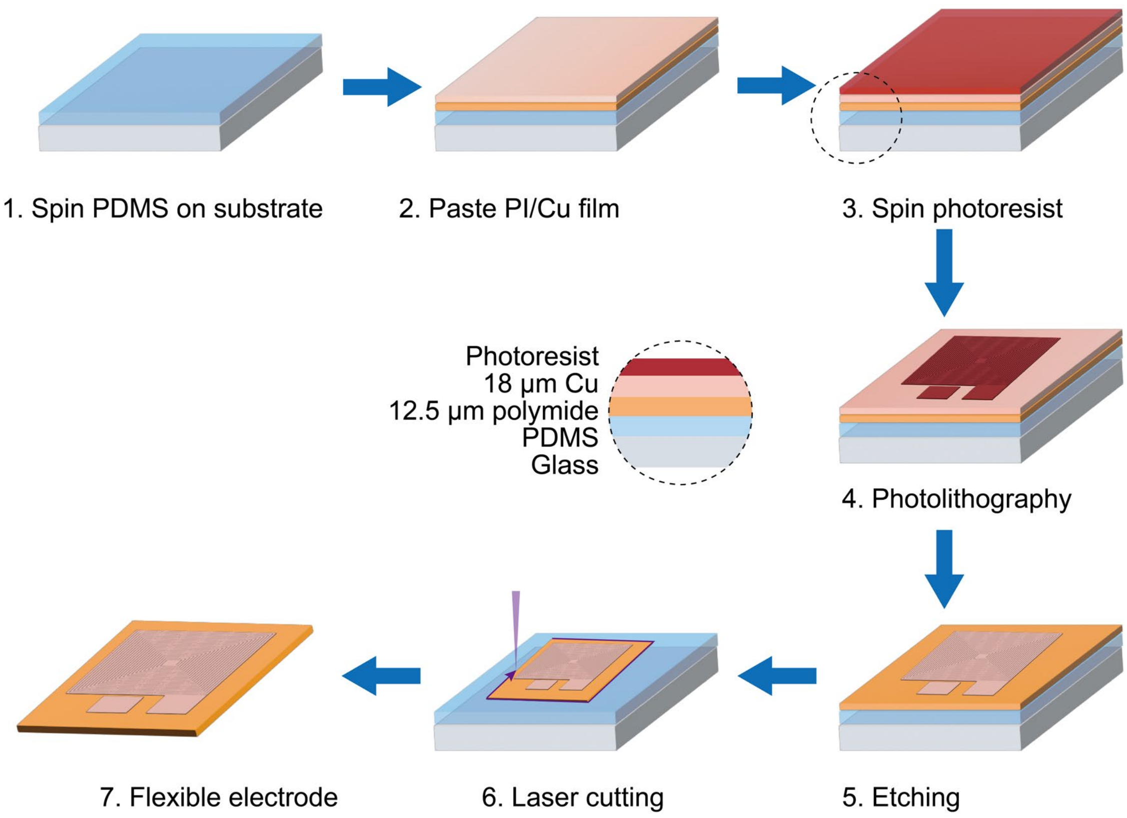

Fig. S1.

Fabrication process of the flexible coil. The main process includes spinning PDMS on the glass substrate, pasting a thin copper-clad polyimide (PI/Cu) film, spinning positive photoresist, photolithography, wet etching, and laser cutting.

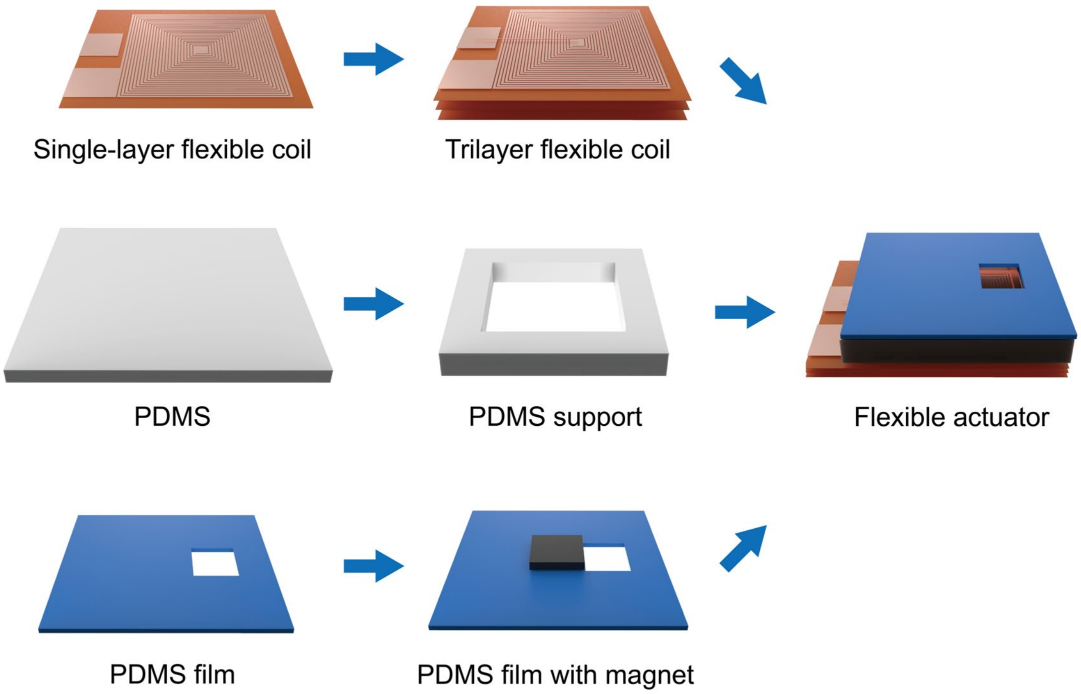

Fig. S2.

Fabrication of the flexible self-sensing actuator. The flexible self-sensing actuator consists of a trilayer flexible coil, a soft PDMS support, a soft PDMS film with a small magnet. The body of the actuator is flexible due to the good flexibility of the individual accessories.

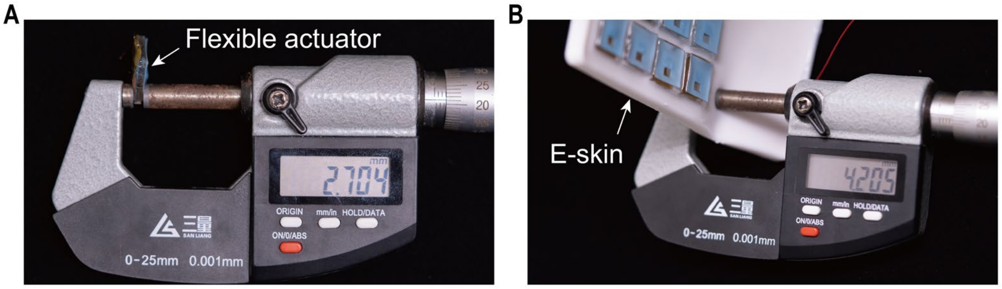

Fig. S3.

Thickness measurement of the flexible self-sensing actuator and the e-skin. (**A**) The thickness of the flexible self-sensing actuator is 2.7mm. (**B**) The thickness of the e-skin is 4.2mm.

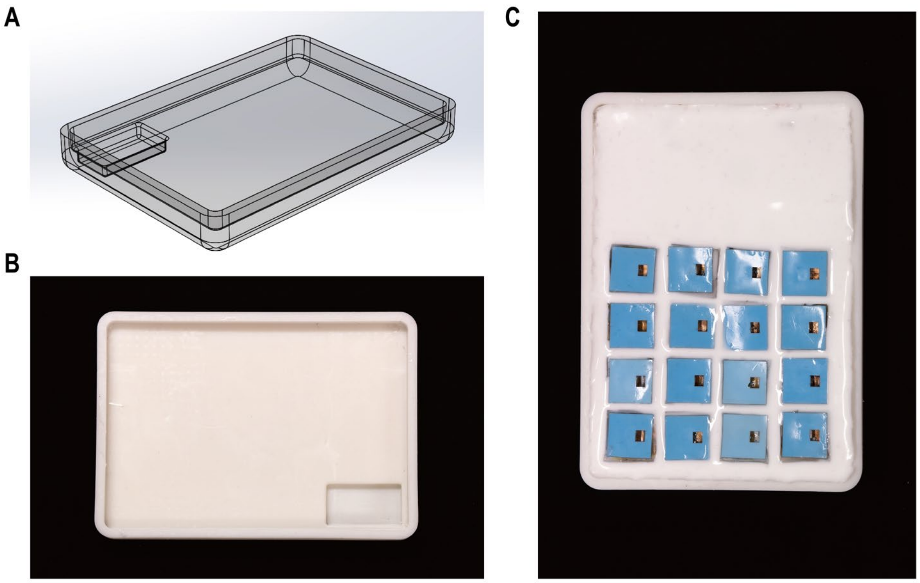

Fig. S4.

Encapsulation of the self-sensing and haptic-reproducing e-skin. (**A**) 3D design drawing of the encapsulation mold. (**B**) 3D printed encapsulation mold coated with parylene C. (**C**) Encapsulated self-sensing and haptic-reproducing e-skin with a 4×4 array of actuators.

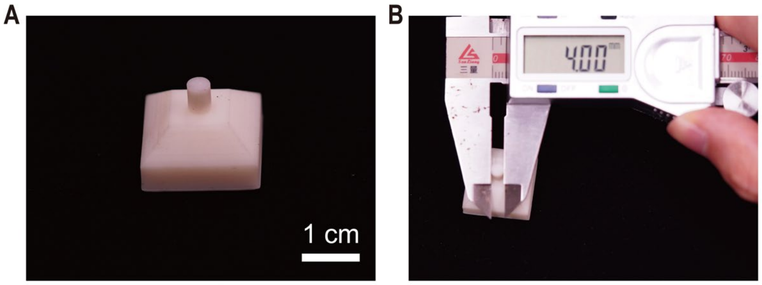

Fig. S5.

Bump block for the tactile sensing test. (**A**) Picture of the bump block. (**B**) Diameter measurement of bump block top. The diameter of the cylinder is 4mm.

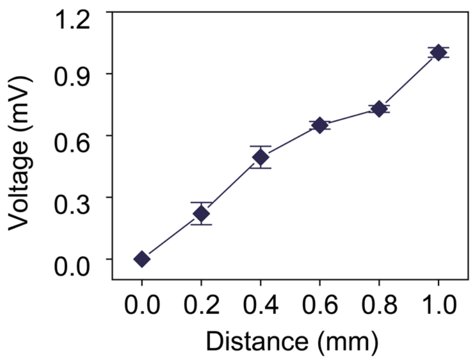

Fig. S6.
Relationship between the sensing voltage and the pressing distance.

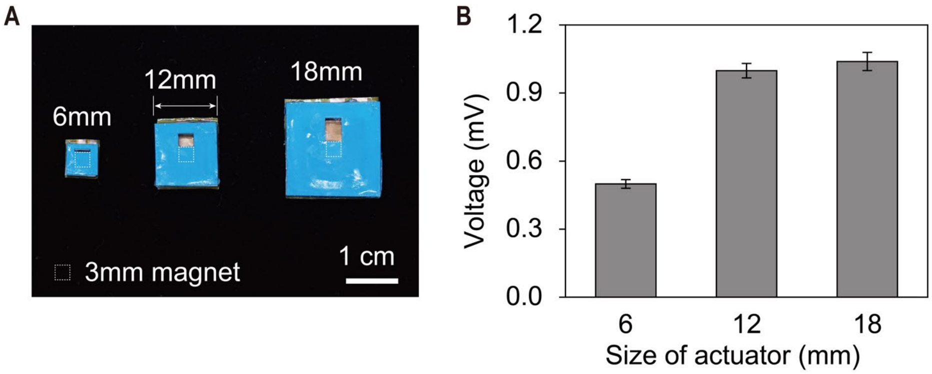

Fig. S7.

Relationship between the sensing voltage and the actuator size with the same magnet. (**A**) Pictures of the actuators with length of 6mm, 12mm and 18mm. The size of the square magnet is 3mm. The sizes of the trilayer coils are 6mm, 12mm and 18mm, respectively. (**B**) Sensing voltages of the different actuator size.

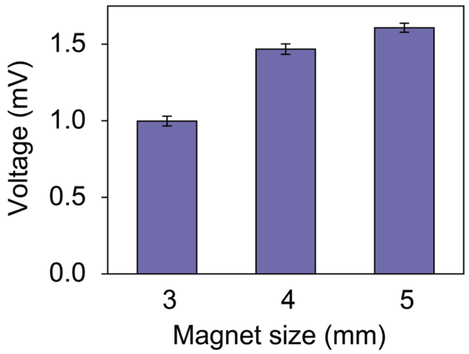

Fig. S8.
Relationship between the sensing voltage and the magnet size for the 12mm actuator.

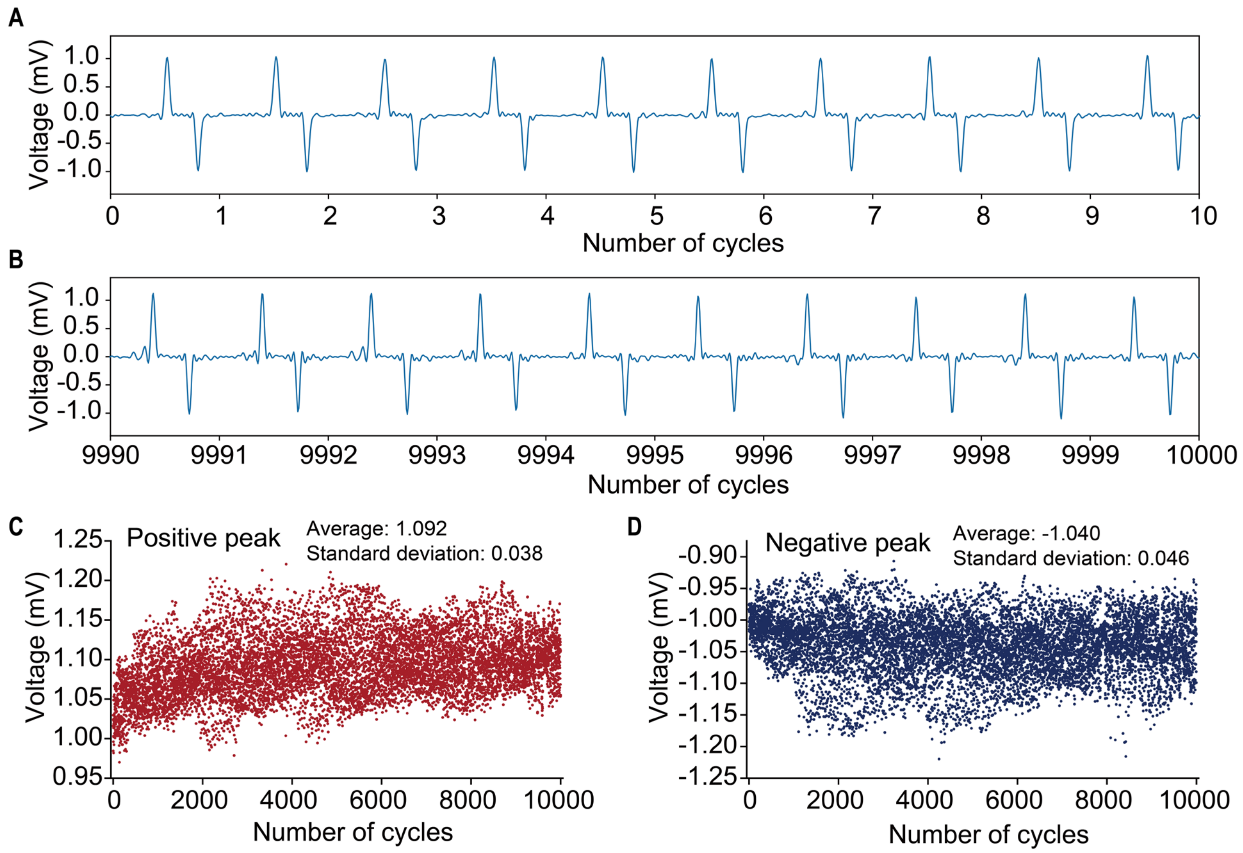

Fig. S9.

Sensing cycle performances of the flexible actuator. (**A**) The sensing voltage from the 1st cycle to the 10th cycle. (**B**) The sensing voltage from the 9990th cycle to the 10000th cycle. (**C**) Distribution of the positive peaks values during the 10000 cycles. (**D**) Distribution of the negative peaks values during the 10000 cycles.

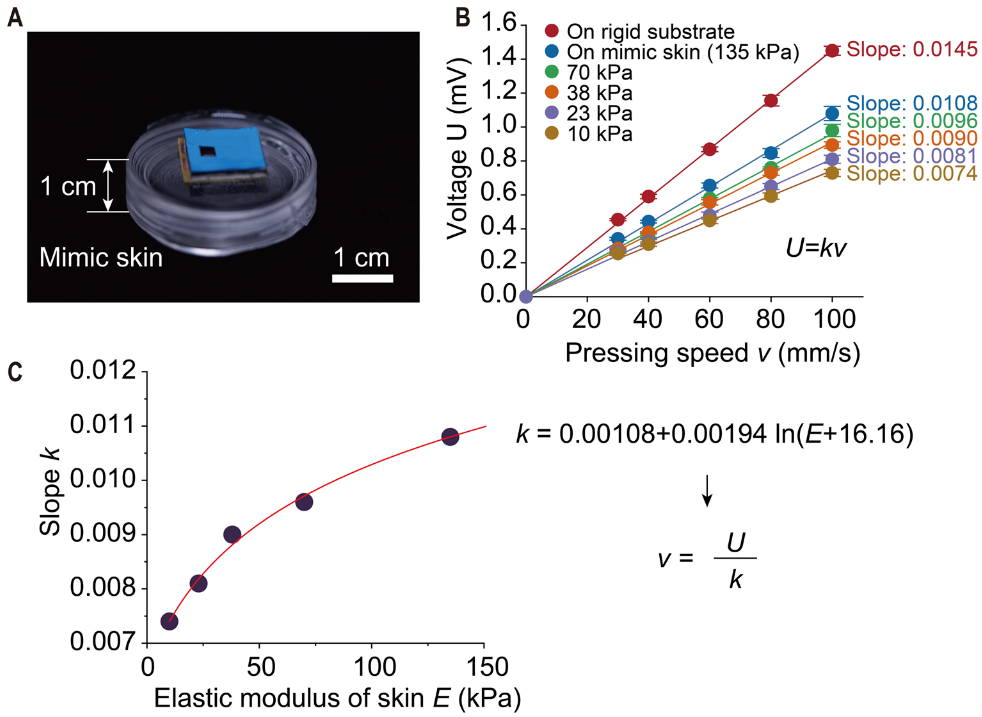

Fig. S10.

Sensing performances of the flexible actuator on the soft mimic skin. (**A**) A flexible self-sensing actuator on the 1cm-thick mimic skin. The soft mimic skin was fabricated from the polydimethylsiloxane (PDMS) with pre-polymer and crosslink agent mass rate of 30:1, 35:1, 40:1, 45:1, 50:1 with elastic modulus of 135kPa, 70kPa, 38kPa, 23kPa and 10kPa, respectively. (**B**) Relationship of sensing voltages of the device and the pressing speed on the rigid substrate and on the 1cm-thick mimic skins with different elastic modulus. (**C**) Relationship of slope in (B) with the elastic modulus of mimic skins. The test was carried out by using the test setup in Fig. 2B. When the device was pressed on the thick soft PDMS, the soft mimic skin deformed and thinned. Then, the device moved away from the bump block as the mimic skin was depressed. The resulting reduction in the distance between the pressed magnet and the device coil will result in a lower sensing voltage. The greater the pressing speed, the greater the reduction in sensing voltage, which also results in a smaller slope of change in sensing voltage with pressing speed on the mimic skin.

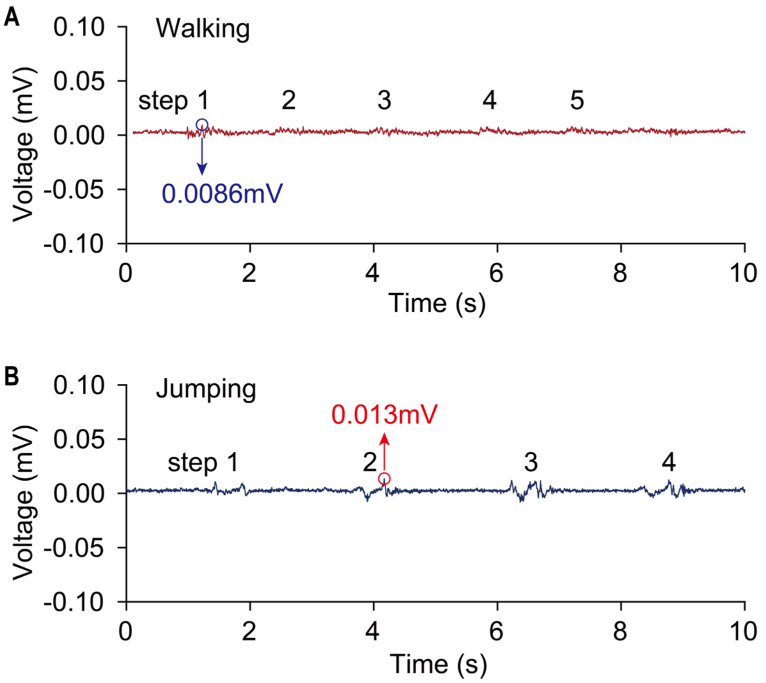

Fig. S11.

Interference testing of sensing signals during daily wear and movement. The actuator was attached on the upper limb during the test. (**A**) Sensing voltage signals of the device during walking. The maximum peak value is only 0.0086mV. (**B**) Sensing voltage signals of the device during jumping. The maximum peak value is only 0.013mV. For daily use, the sensing voltage is bigger than 0.3mV. The sensing signal generated by body movement can be ignored as it is much smaller than the sensing signal from touch.

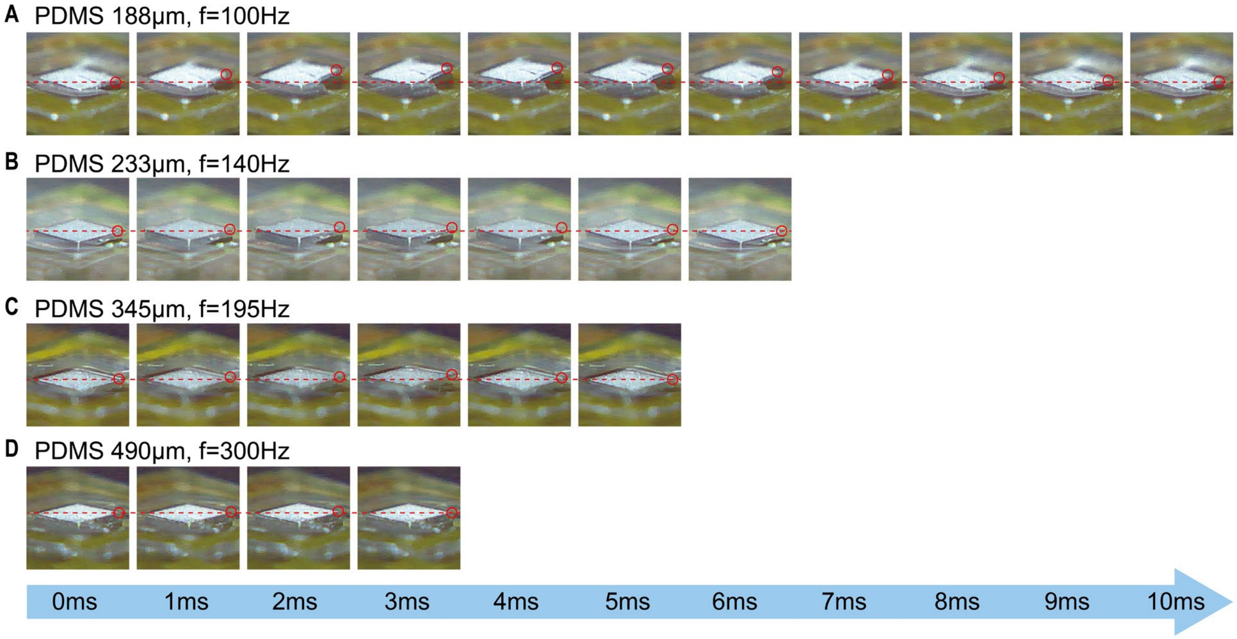

Fig. S12.

Vibration behaviors of the actuators with different PDMS thickness. A sinewave current with value of 50 mA was applied on the actuators. For the actuators with 188μm-, 233μm-, 345μm- and 490μm-thick PDMS film, their resonant frequencies are 100Hz (**A**), 140Hz (**B**),195Hz (**C**), and 300Hz (**D**), respectively.

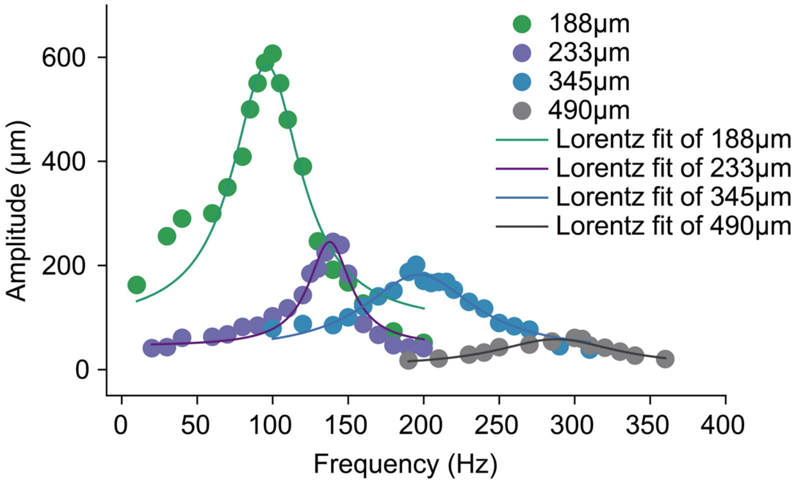

Fig. S13.

Relationship between vibration amplitude and frequency under 50mA for the actuators with different PDMS thickness. For the actuators with 188µm-, 233µm-, 345µm-, 490µm-thick PDMS film, the corresponding vibration amplitudes are 606.7µm at 100Hz, 245.3µm at 140Hz, 201.3µm at 195Hz, and 60.6µm at 300Hz, respectively. The thicker the PDMS film is, the higher the resonant frequency of the actuator is. Under the same actuation current, the larger the resonant frequency is, the smaller the amplitude at the resonant frequency is.

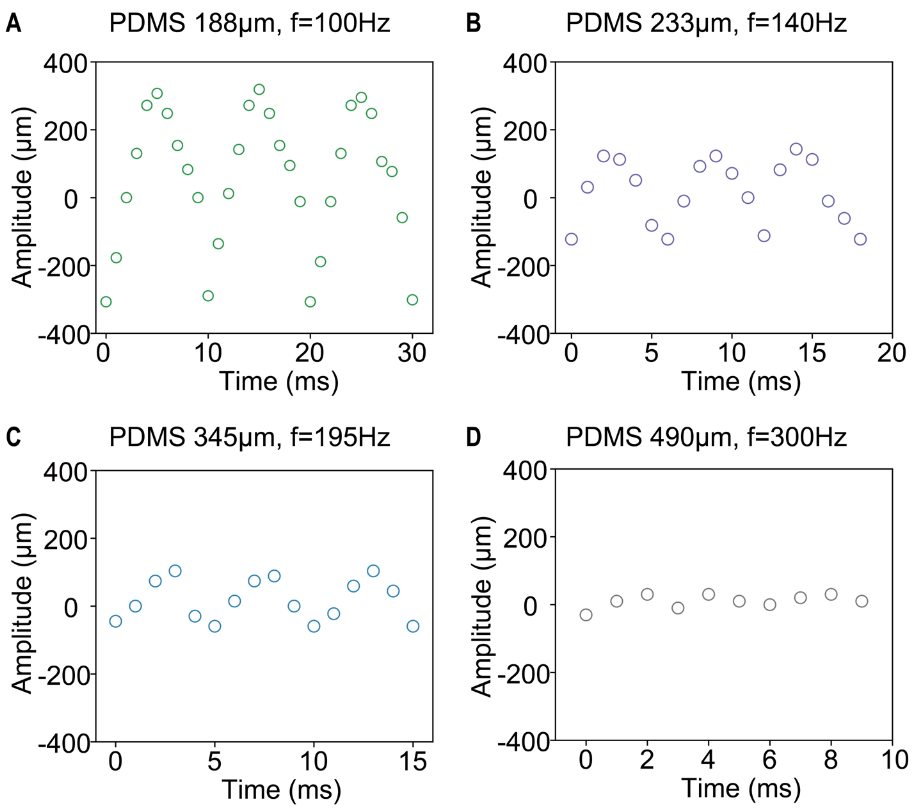

Fig. S14.

Variation amplitude with time at resonant frequencies for the actuators with different PDMS thickness. (**A**) A 50mA sinewave current with frequency of 100Hz was applied on the actuator. (**B**) A 50mA sinewave current with frequency of 140Hz was applied on the actuator. (**C**) A 50mA sinewave current with frequency of 195Hz was applied on the actuator. (**D**) A 50mA sinewave current with frequency of 300Hz was applied on the actuator.

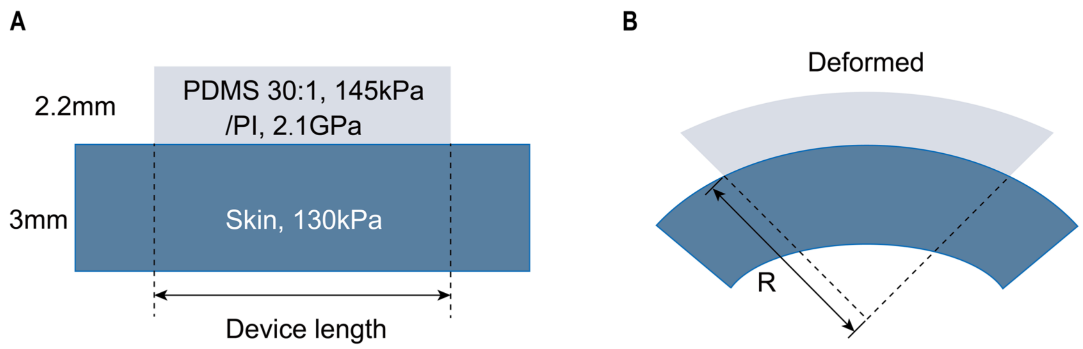

Fig. S15.

Schematic illustration of the simplified actuator attached to human skin applied bending deformation. (**A**) Cross-section view of the simplified actuator (PDMS or PI) attached to the skin surface and their associated Young's modulus. (**B**) Cross-section view of the simplified device attached to skin under the bending deformation with a curvature radius of R.

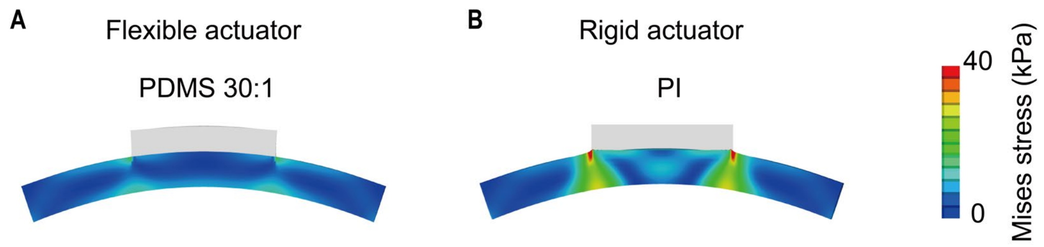

Fig. S16.

The influence of the Young's modulus of the simplified actuator on the Mises stress distributions on the skin. The Mises stress distributions on the bending skin (R=28 mm) with the flexible actuator (**A**) and rigid actuator (**B**), respectively. See Fig. S15 for the details of the model.

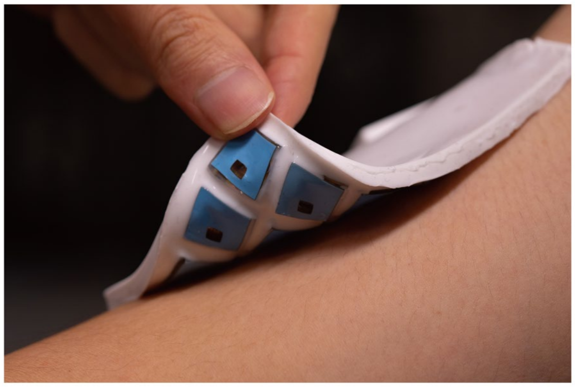

Fig. S17.

A self-sensing and haptic reproducing e-skin on the skin. The soft and thin e-skin presents a good fit to the skin.

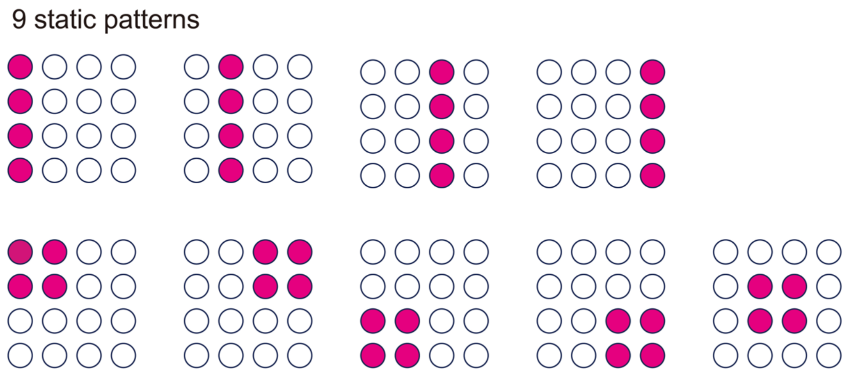

Fig. S18.
Nine static patterns for haptic feedback test.

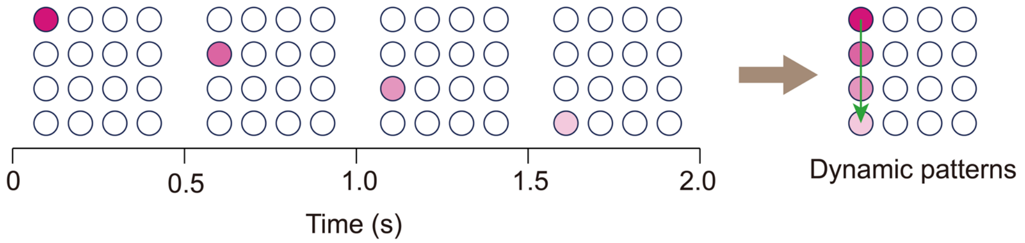

Fig. S19.

The actuation process of the dynamic haptic feedback patterns in the user study. Each actuator ran for 0.5s and was then switched sequentially.

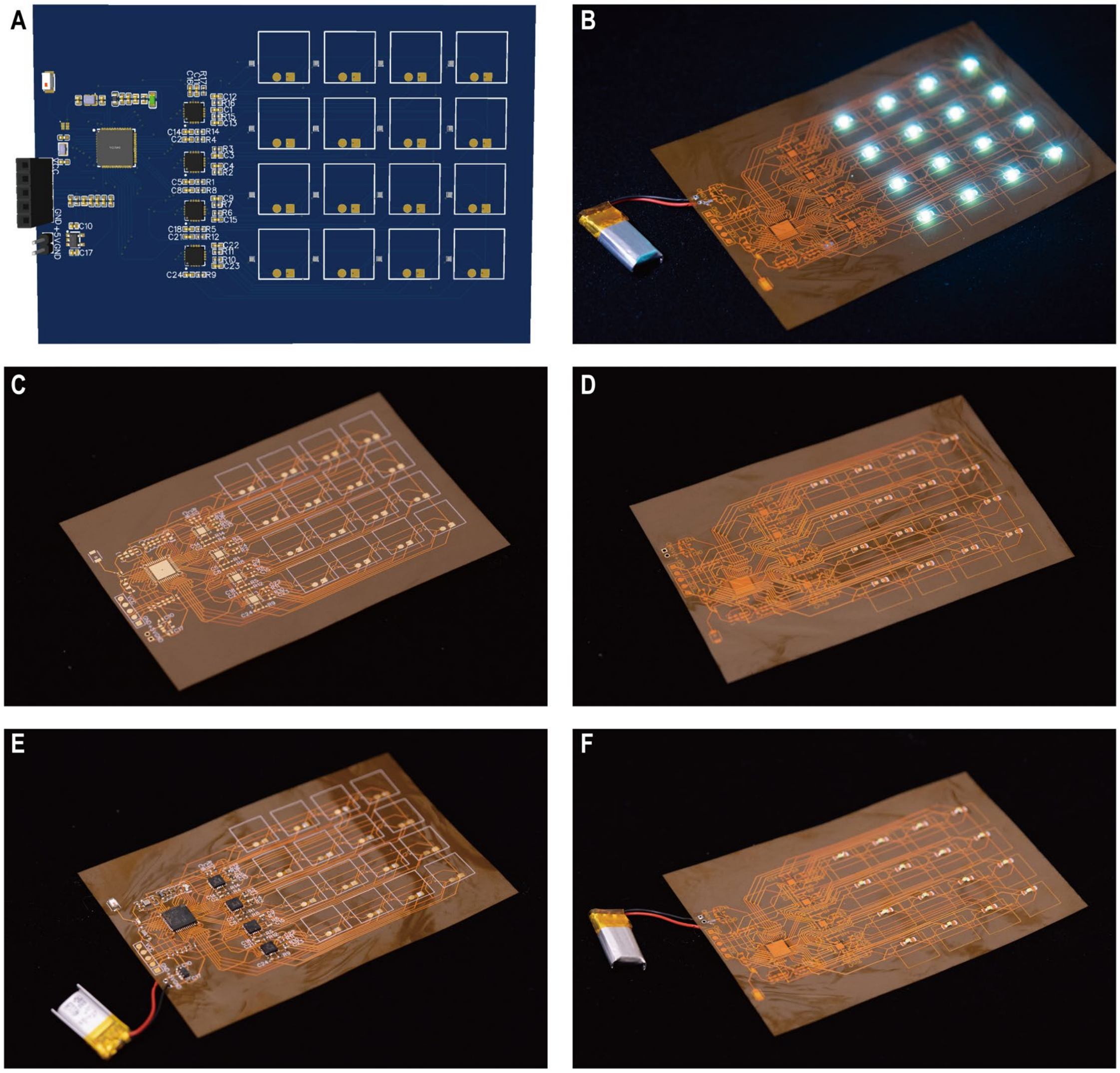

Fig. S20.

Wireless circuit modules for the self-sensing and haptic-reproducing e-skin. (**A**) The design of the circuit boards. (**B**) A thin flexible printed circuit board (FPCB) with all LEDs lighting up. (**C**) Front side of the FPCB. (**D**) Back side of the FPCB. (**E**) Front side of the FPCB with fully soldered components including capacitors, resistors, chips, electronic switches, etc. (**F**) Back side of the FPCB with fully soldered LEDs.

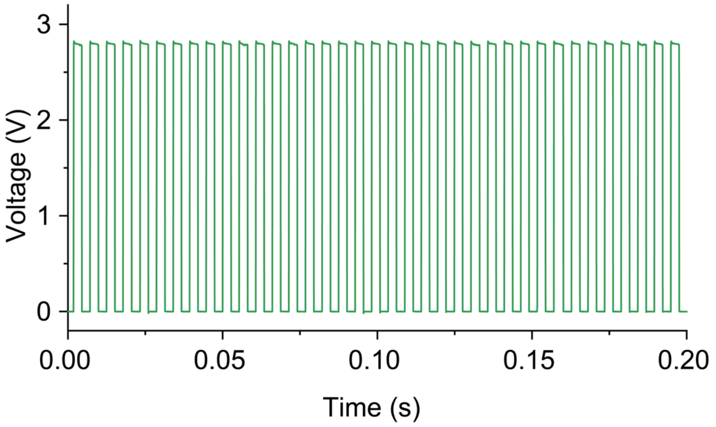

Fig. S21.

Actuation voltage to the actuator supplied by the wireless circuit. The voltage is measured with the actuator as a load. The AC square-wave voltage with a high-level value of 2.8V, a low-level value of 0V and a frequency of 195Hz was applied to drive the actuators with 345μm-thick PDMS film.

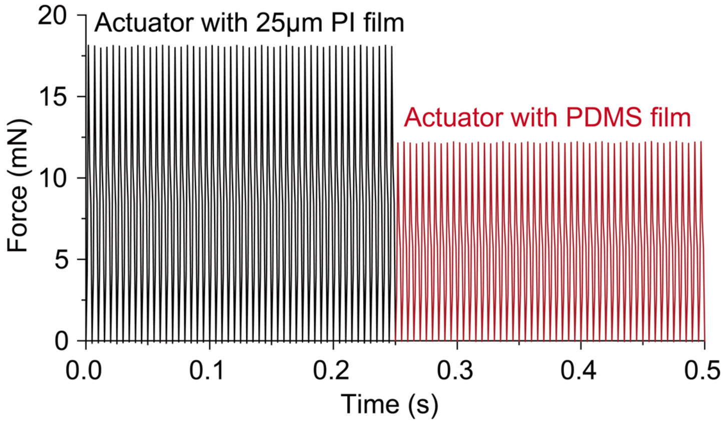

Fig. S22.

Feedback force of the actuators with powered by the flexible circuit. (Left) Actuator with the 25μm-thick polyimide (PI) film with elastic modulus of 2.1GPa. (Right) Actuator with the 345μm-thick PDMS film with elastic modulus of 3MPa.

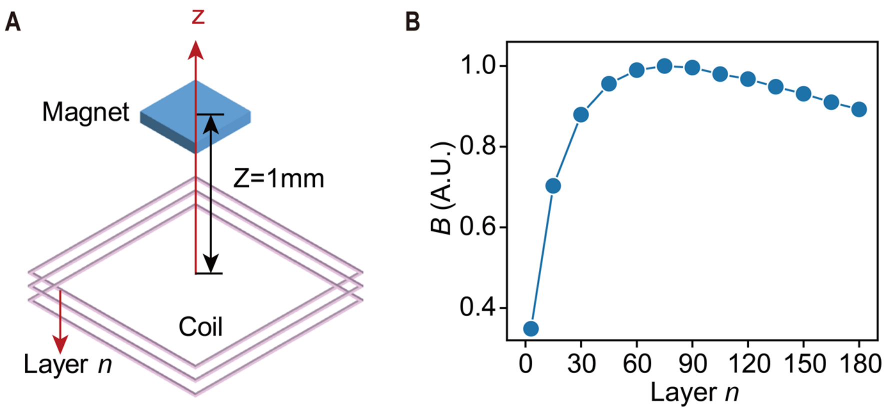

Fig. S23.

Simulation of the magnetic flux density of the coil with different layers. (A) Schematic illustration of the coil with different number of layers in the actuator. (B) Relationship between the simulated normalized magnetic flux density (B) and the number of the coil layer (n). The actuation power was set as a constant for the coil with different layers. The coil with the maximum magnetic flux density is 75 layers corresponding to 1725 turns.

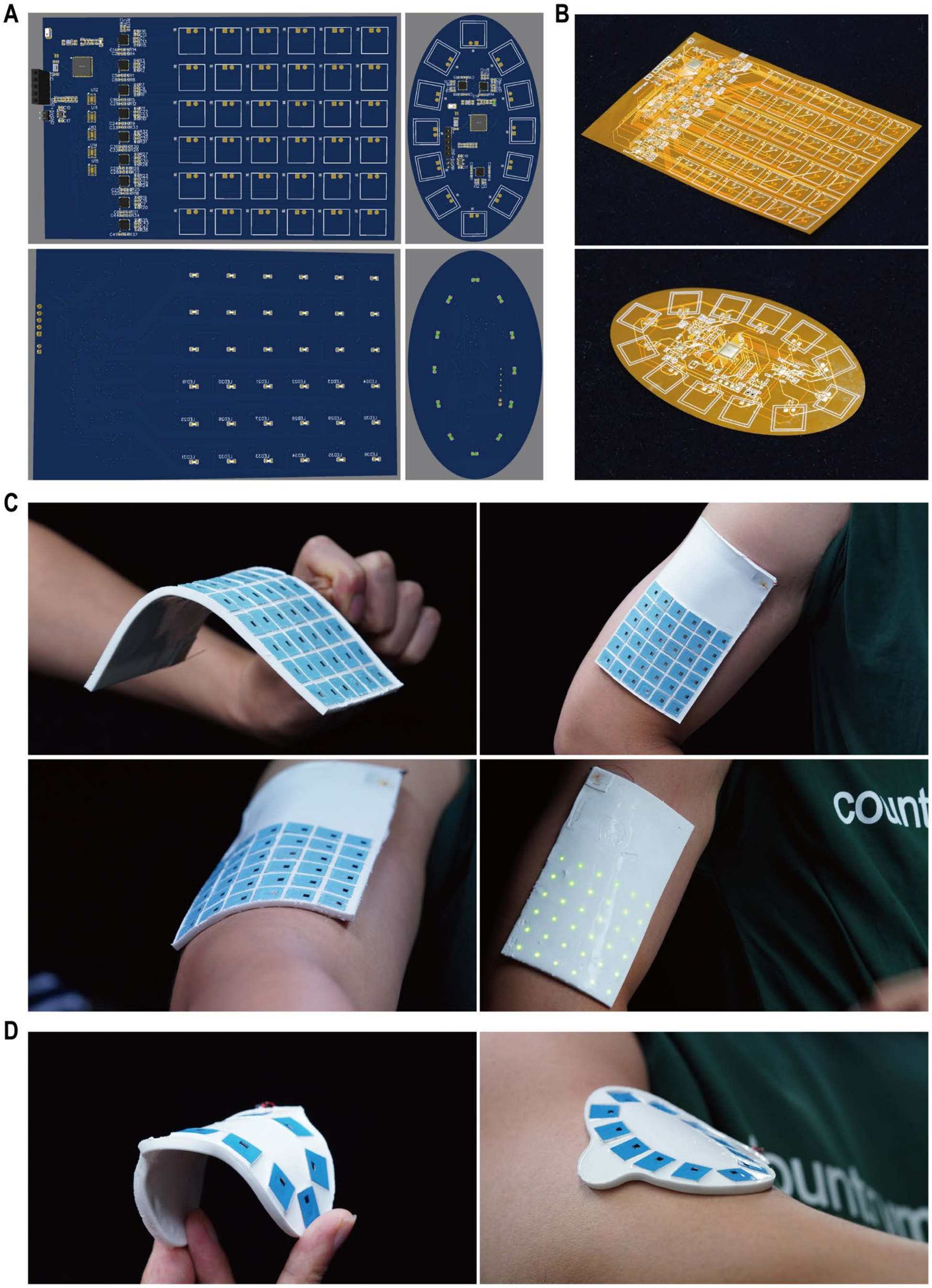

Fig. S24.

Scalable design of the self-sensing and haptic-reproducing e-skin. (**A**) The design of the circuit boards. The left one is a rectangular board with a 6×6 actuators array, and the right one is an elliptical shaped board with 12 actuators. (**B**) The flexible printed circuit board (FPCB) for these two scalable e-skins. (**C**) Pictures of the e-skin with 6×6 actuators array attached to the skin. (**D**) Pictures of the e-skin with an elliptical shape and 12 actuators.

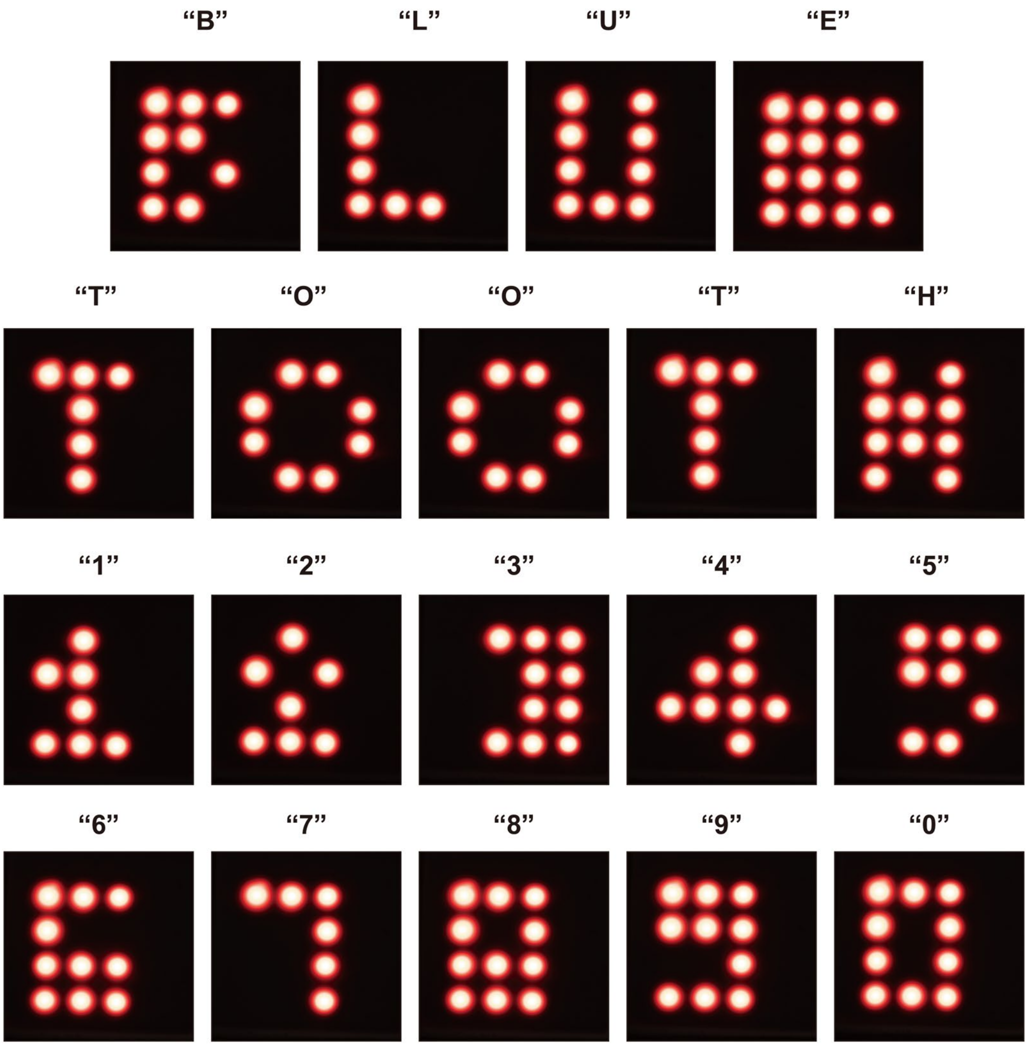

Fig. S25.

Actuation patterns for the self-sensing and haptic-reproducing e-skin. On the 4×4 actuators array, the programmable patterns were actuated with corresponding LEDs lighting up to show different letters and numbers. The letters 'B', 'L', 'E', 'T', 'O', 'O', 'T' and 'H' were actuated to form the word "BLUETOOTH". The numbers '1', '2', '3', '4', '5', '6', '7', '8', '9' and '0'were displayed.

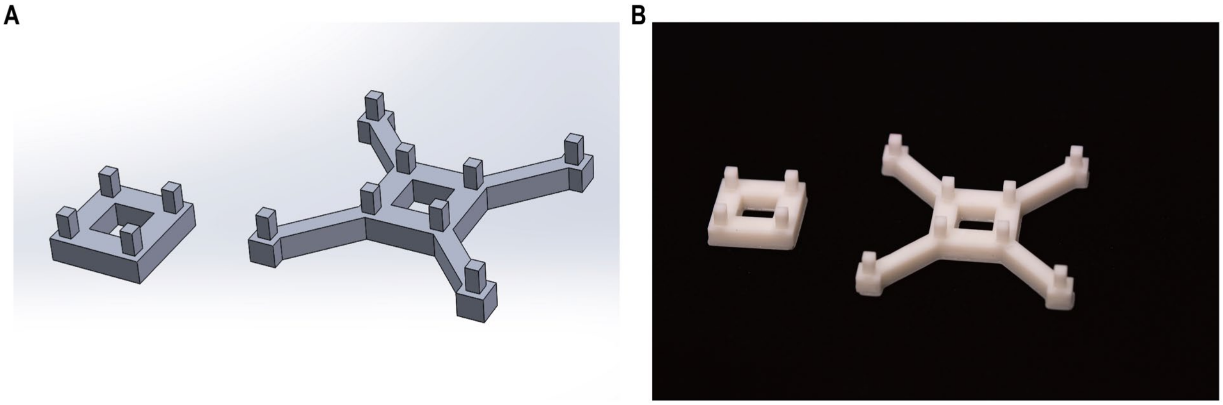

Fig. S26.

Printed blocks for the sensing presses on the self-sensing and haptic-reproducing e-skin. (**A**) 3D design drawing for the blocks. (**B**) 3D printed block.

Movie S1.

Periodic oscillation of a haptic actuator based on electromagnetic simulation.

Movie S2.

Vibration behaviors of the flexible self-sensing actuators with different PDMS thickness. For the actuators with 188μm-, 233μm-, 345μm-, 490μm-thick PDMS film, the corresponding resonant frequencies are 100Hz, 140Hz, 195Hz and 300Hz, respectively. A sinewave AC current with value of 50 mA was applied on these actuators. The thicker the PDMS is, the higher the resonant frequency of the actuator is.

Movie S3.

FEA results of the vibrational state for an actuator mounted on (left) undeformed skin and (right) bending deformation skin with curvature radius of R=28mm, where U represents the normalized displacement amplitude.

Movie S4.

Actuation patterns of "TOUCH-IoT" for the self-sensing and haptic-reproducing e-skin. The programmable patterns were actuated with corresponding LEDs lighting up to show different haptic feedback patterns in letters and symbol 'T', 'O', 'U', 'C', 'H', '-', 'I', 'o' and 'T'.

Movie S5.

One-to-one bi-directional wireless touch intercom. In two different rooms, the touch is transmitted wirelessly in both directions based on the self-sensing and haptic reproduction functions of the e-skin.

Movie S6.

Wireless touch IoT. A touch IoT is built based on multiple e-skins. Touch can be transmitted wirelessly in both directions between any number of these e-skins.
